# Efficiency of ddRAD target enriched sequencing across spiny rock lobster species (Palinuridae: *Jasus*)

**DOI:** 10.1038/s41598-017-06582-5

**Published:** 2017-07-28

**Authors:** Carla A. Souza, Nicholas Murphy, Cecilia Villacorta-Rath, Laura N. Woodings, Irina Ilyushkina, Cristian E. Hernandez, Bridget S. Green, James J. Bell, Jan M. Strugnell

**Affiliations:** 1 Department of Ecology, Environment and Evolution, School of Life Sciences, La Trobe University, Melbourne, VIC 3086 Australia; 2 Institute for Marine and Antarctic Studies, University of Tasmania, Hobart, TAS 7001 Australia; 3 School of Biological Sciences, Victoria University of Wellington, Wellington, 6140 New Zealand; 4 Departamento de Zoologia, Facultad de Ciencias Naturales y Oceanograficas, Universidad de Concepcion, Concepcion, Chile; 5 Centre for Sustainable Tropical Fisheries and Aquaculture and College of Science and Engineering, James Cook University, Townsville, QLD 7001 Australia

## Abstract

Double digest restriction site-associated DNA sequencing (ddRADseq) and target capture sequencing methods are used to explore population and phylogenetic questions in non-model organisms. ddRADseq offers a simple and reliable protocol for population genomic studies, however it can result in a large amount of missing data due to allelic dropout. Target capture sequencing offers an opportunity to increase sequencing coverage with little missing data and consistent orthologous loci across samples, although this approach has generally been applied to conserved markers for deeper evolutionary questions. Here, we combine both methods to generate high quality sequencing data for population genomic studies of all marine lobster species from the genus *Jasus*. We designed probes based on ddRADseq libraries of two lobster species (*Jasus edwardsii* and *Sagmariasus verreau*xi) and evaluated the captured sequencing data in five other *Jasus* species. We validated 4,465 polymorphic loci amongst these species using a cost effective sequencing protocol, of which 1,730 were recovered from all species, and 4,026 were present in at least three species. The method was also successfully applied to DNA samples obtained from museum specimens. This data will be further used to assess spatial-temporal genetic variation in *Jasus* species found in the Southern Hemisphere.

## Introduction

Target enriched, or target-capture next-generation sequencing, has been successfully applied to assess genome-scale data in non-model species lacking a reference genome. The method uses 60- to 120-mer probes for in-solution hybridization to capture specific genome targets for sequencing. This approach has proven to be useful in addressing phylogenetic questions (reviewed by McCormack^1^) and has been employed in studies incorporating museum specimens^2–6^. However, the design of probes for target capture methods requires high-quality genomic or transcriptomic resources, which limits its application to a wide range of taxa^7, 8^. Loci obtained using most capture methods to date are either subject to selection (e.g. in the case of exon derived transcriptomic markers) or are highly conserved within species (e.g. for probes that use ultra-conserved elements). Therefore, capture-based approaches have not been widely used for studying population-level processes such as genetic drift and gene flow, and have had limited application to population genetic studies.

The majority of population genomics studies have been undertaken using restriction digest derived methods (e.g. RADSeq, GBS, ddRAD). These methods comprise a range of related protocols, employing restriction digestion and library size selection in order to reduce genome complexity, enabling the study of genome-wide genetic variation without any prior genomic knowledge^9–11^. However, issues such as PCR duplication bias, large amounts of missing data due to allelic dropout, low reproducibility and the requirement of high molecular weight DNA have been reported from these studies^12–15^. The main shortcomings of this approach are the difficulties of cross species comparison^16^ and repeatability of sequencing across multiple libraries, even of replicate samples. Additionally, poor quality DNA means that the application of restriction digest-based approaches is mostly unsuitable for preserved material (e.g. museum collections).

Recently, methods combining the benefits of RADseq (e.g. no requirement for genomic resources) and target-capture (e.g. repeatability across samples) approaches have been developed to improve sequencing coverage in non-model organisms. Examples of these protocols include HyRAD^5^, RADcap^17^ and Rapture^18^, and although similar, each technique offers distinct benefits and limitations. HyRAD supports the use of highly degraded DNA samples and offers cost-effective benefits such as the use of ‘home-made’ probes (biotinylated ddRAD libraries); the Rapture protocol produces the highest coverage from minimal reads per individual; RADcap has proven to be cost-effective and uses adapters with degenerated bases in ddRAD libraries enabling identification of PCR duplicates. These methods all offer promise for combining the efficiency and repeatability of capture-based approaches with the utility for population genomics studies of non-model species. Here we report on a similar, yet alternate approach for restriction-based capture libraries and assess the utility of this approach for multiple species population genomics studies, and, in particular for incorporating museum samples into these studies.

In this study we used existing ddRADseq sequence databases of two closely related marine lobster species, *Jasus edwardsii* ^15^ (N = 42) and *Sagmariasus verreauxi* (N = 55), to design probes for target enrichment re-sequencing. We then evaluated the efficiency and reproducibility of the target enrichment approach, and determined the practical utility of this method for population genomic studies within all marine spiny lobster species of the genus *Jasus* in modern (N = 40) and museum collection (N = 39) specimens. We implemented a modified target-capture protocol to: (1) generate cost effective, high quality population genomic data across *Jasus* species using a single target panel; (2) reduce the amount of missing data reported from previous ddRAD experiments; and (3) recover consistent sequencing data from highly degraded museum collection samples by using high confidence probabilistic base-calling.

## Results and Discussion

### ddRAD loci catalogue and probe design

**Figure 1:**
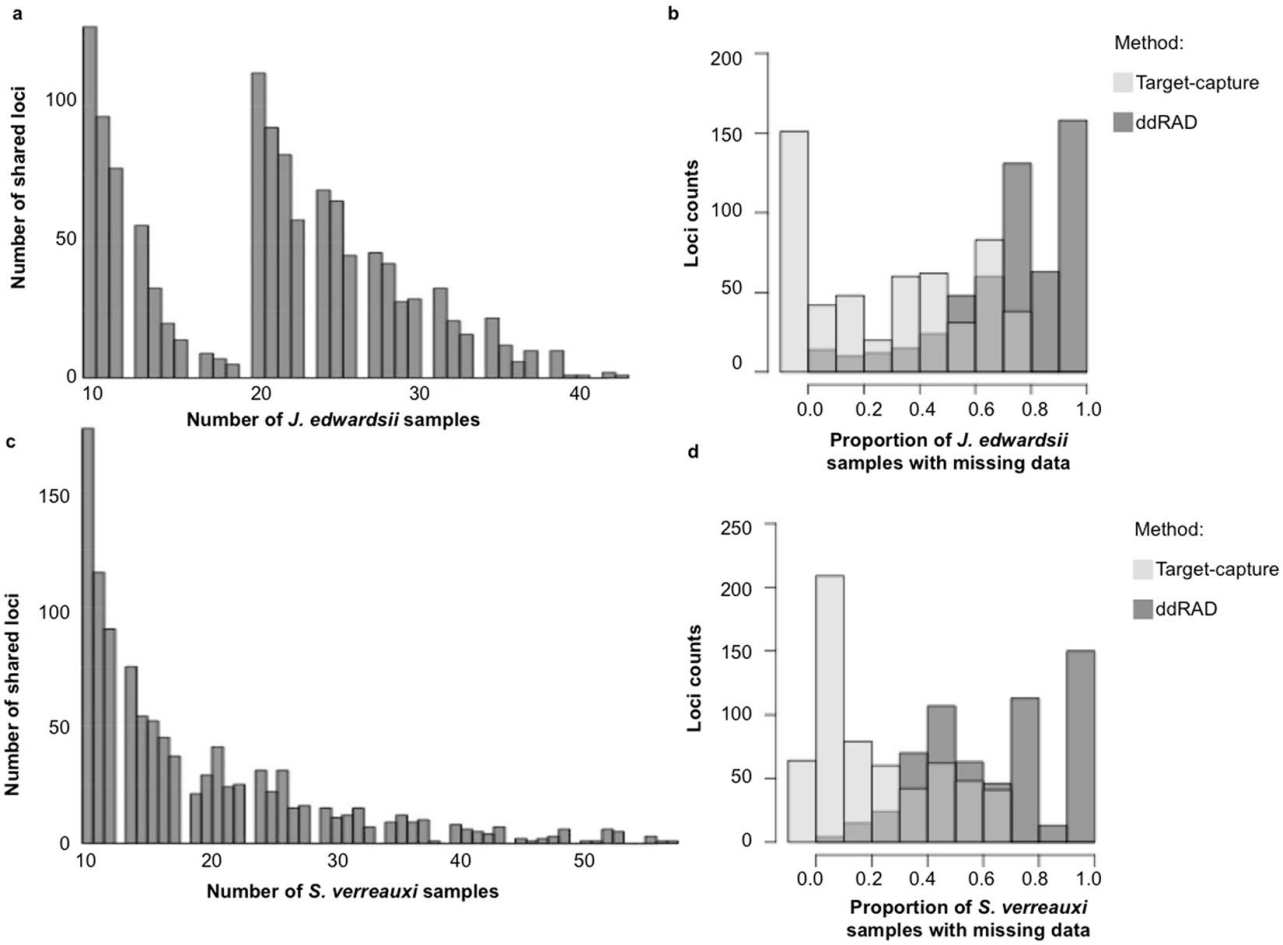
Histograms displaying the number of shared loci among samples in ddRAD libraries of *J. edwardsii* (**a**) and *S. verreauxi* (**c**) samples. Overlapping histograms of target-capture and ddRAD representing the percentage of missing data among validated loci in *J. edwardsii* (**b**) and *S. verreauxi*i (**d**).

The *J. edwardsii* ddRAD libraries published by Villacorta-Rath *et al*. (2016)^15^, and *S. verreauxi* libraries generated in this work were first assembled in PyRAD^19^ in order to obtain a ddRAD loci catalogue. This was used as a template to design the MYbait^®^ probes. Three PyRAD assemblies (*J. edwardsii*, *S. verreauxi* and a combined species assembly) resulted in a catalogue of 4,629 loci (Supplementary Methods S1). *De novo* assembly, using liberal similarity thresholds (75%), revealed putative paralogous loci, which were discarded. Redundant loci (present in multiple assemblies) were synonymized into single loci as described in the Methods section. Across all three assemblies only 123 loci were shared between the two species, and 2,267 were species-specific (1,219 were from *J. edwardsii* and 1,048 were from *S. verreauxi)*. The percentage of missing loci over all samples was 52.4 ± 19.2 for *J. edwardsii* and 66.3 ± 18.3 for *S. verreauxi* (Fig. 1a and c). After discarding putative paralogous loci and simple repeat regions, 2,366 ddRAD loci were selected as templates for manufacturing MYbaits^®^ probes.

### Target enriched sequencing efficiency

MYbait^®^ probes were used to capture 2,366 ddRAD loci from 87 individuals, representing the six *Jasus* species (47 obtained from museum samples) and 16 individuals of *S. verreauxi* (Table 1). The enriched loci were sequenced on a single 250 X 2 Illumina MiSeq sequencing run, generating 4.6 Gbp (giga base pairs) of sequence data (1.29% sequencing error rate and 84.49% of data above Q30 score). Subsequent trimming and removal of low quality reads and external contaminants resulted in 800 and 174 Mbp high-quality data for modern and museum specimens, respectively. The lower sequencing output for museum samples is likely due to low yield DNA extracts, which were highly fragmented and possessed a low A_260_/A_280_ ratio (Supplementary Fig. S2). For example, none of the 50-year-old *J. frontalis* museum samples (N = 8) generated sequencing data sufficient to enable variant calling and were excluded from downstream analysis.

**Table 1:** Sampling localities, species designations and number of individuals sampled (N).

Species	Country of Origin	Location	Date	N
*J. edwardsii*	Australia	Victoria, West, North, South of Tasmania and Tasman Sea	1991	8
	Australia	Victoria, Southwest Tasmania and East Tasmania	2013–2014	8
*J. caveorum*	British Overseas Territory	South East Pacific, Pitcairn Island, Foundation Seamounts	1995	7
*J. frontalis*	Chile	Juan Fernandez Archipelago, Islas Desventuradas	1967	8
	Chile	Juan Fernandez Archipelago, Islas Desventuradas	2010	8
*J. lalandii*	Namibia/South Africa	WestSouthern Africa	1991	8
	South Africa	Cape Town	1967	8
	South Africa	East coast and islands	2015	8
*J. paulensis*	French Overseas Territory	Saint Paul and Amsterdam Islands	1967	8
	French Overseas Territory	Saint Paul and Amsterdam Islands	2014	8
*J. tristani*	South Africa	Tristan da Cunha Seamount	2015	8
*S. verreauxi*	Australia	Tasmania, New South Wales	2013	8
	New Zealand	Cape Maria Van Diemen to North Cape	2013	8
Total				103

### Probe efficiency for *J. edwardsii* and *S. verreauxi*

As the probes were developed with *J. edwardsii* and *S. verreauxi* ddRAD libraries, we initially examined the probe capture efficiency in these two taxa.

#### ddRAD loci recovered

The number of reads recovered that were directly BLAST^20^ matched to the designed probes varied from 72.69% in *J. edwardsii* and 76.52% in *S. verreauxi*, covering 2,045 ddRAD loci by at least one sequence read (Table 2). We found 721 loci shared between species and 1,324 were species-specific (795 in *J. edwardsii* and 529 in *S. verreauxi*). A lower percentage of reads were correctly mapped using Bowtie 2^21^ aligner (55.04% in *J. edwardsii* and 57.52% in *S. verreauxi*) and fewer loci (1,250 loci) were mapped to the probe set with mapping quality greater than five (maQ ≥ 5), of which 456 were shared between species (Table 2). Compared to the initial ddRAD sequencing (123 shared loci) the target-capture method resulted in a four-fold increase in shared markers between these genera, including a number of loci identified as putatively under selection by Villacorta-Rath *et al*.^15^. In addition, the target-capture approach resulted in less missing data within each species than the ddRAD sequencing approach (Fig. 1b and d). This is most likely due to allele dropout in ddRAD libraries usually caused by polymorphisms in the restriction sites, which has the potential to bias population genetics analyses^22, 23^ or render them completely unusable.

**Table 2:** Target-capture enriched sequencing and genotyping efficiency between *J. edwardsii* and *S. verreauxi* sampling.

Species	*J. edwardsii* (N = 11)	*S. verreauxi* (N = 16)
Processed reads	740,745	544,149
On-target reads	558,595	416,393
Reads mapped to original probe set	296,247	239,490
PCR duplicates	117,566	91,787
ddRAD loci BLAST hits	1,516	1,250
ddRAD loci mapped (Bowtie2)	922	784
Average coverage	29.21X	19.10X
% missing ddRAD loci*	24.04 ± 19.53	31.20 ± 27.30

*Missing data was estimated as the average missing percentage across validated loci.

We were able to re-sequence and validate 54 loci reported in Villacorta-Rath *et al*.^15^ in eight *J. edwardsii* samples replicates. Besides, the average level of missing data among these loci in our target-capture experiment was 15.96% within *J. edwardsii* species. It means that the ddRAD loci outliers discovered in a previous study^15^ were successfully re-sequenced and could be enriched and sequenced in range of populations to investigate genome signatures of selection.

### Probe efficiency in non-target *Jasus* species (N = 40)

To assess the probe efficiency in non-target species (i.e. those not used for the initial probe design), we assessed the recovery of loci across the modern *Jasus* samples including *J. edwardsii*, *J. frontalis*, *J. lalandii*, *J. paulensis* and *J. tristani*.

#### ddRAD loci recovered

**Figure 2:**
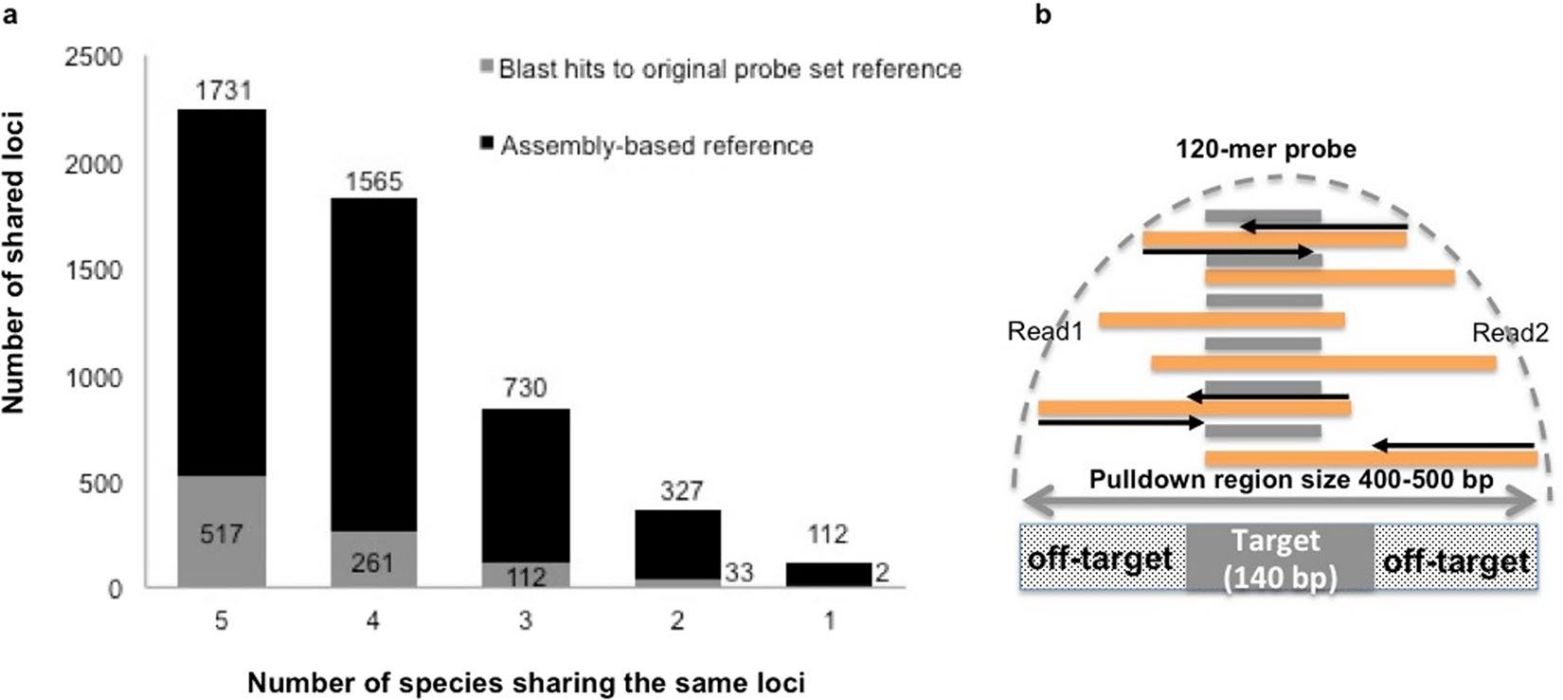
Number of variable loci shared amongst each of six *Jasus* species. (**a**) The left panel displays the number of loci shared among five (5), four (4), three (3) and two (2) species, or found only in a single species (1) in the assembly-based reference (black and grey stacked column) and the corresponding significant BLAST hits to the original probe set (grey). (**b**) The right panel is a graphic representation of target enrichment hybridization reaction showing library size, captured fragments size and off-target reads. The grey lines represent the 120-mer probes, the orange lines represent the library fragments and the black arrows represent overlapping/non-overlapping paired-end reads (Read 1 and Read 2).

Reads mapped to the original probe set using Bowtie 2 covered 925 target loci, varying from 565 in *J. frontalis* to 910 in *J. edwardsii* (Table 3). A total of 517 loci were shared among the five *Jasus* species and a further 112 were shared across at least three species. Only two loci were detected from a single species (Fig. 2a).

**Table 3:** Number of reads mapped to original probe set (ddRAD loci) and assembly-based reference with corresponding levels of similarity threshold (*).

Species	N	Processed reads	Mapped reads to original probe set (%)	PCR duplicates (% over mapped reads)	Blast hits to original probe set**	Mapped reads to new assembly (%)
*J. edwardsii*	*8*	605,396	242,538 (40.06)	97,952(40.39)	910	331,505(42.91)
*J. frontalis*	8	286,288	28,360(9.91)	5,403 (19.05)	565	68,374(15.85)
*J. lalandii*	8	285,545	106,425(37.27)	22,283(20.94)	854	139,969(39.15)
*J. paulensis*	8	478,098	190,903(39.93)	58,824 (30.81)	875	264,249(52.33)
*J. tristani*	8	246,252	94,839(38.51)	24,486(25.82)	829	132,244(31.10)
Total	40	1,901,579	663,065(34.86)	208,948 (31.51)	925*	936,241(49.23)

*Overall loci counts across species. **(maQ ≥ 5).

#### SNP diversity

**Figure 3:**
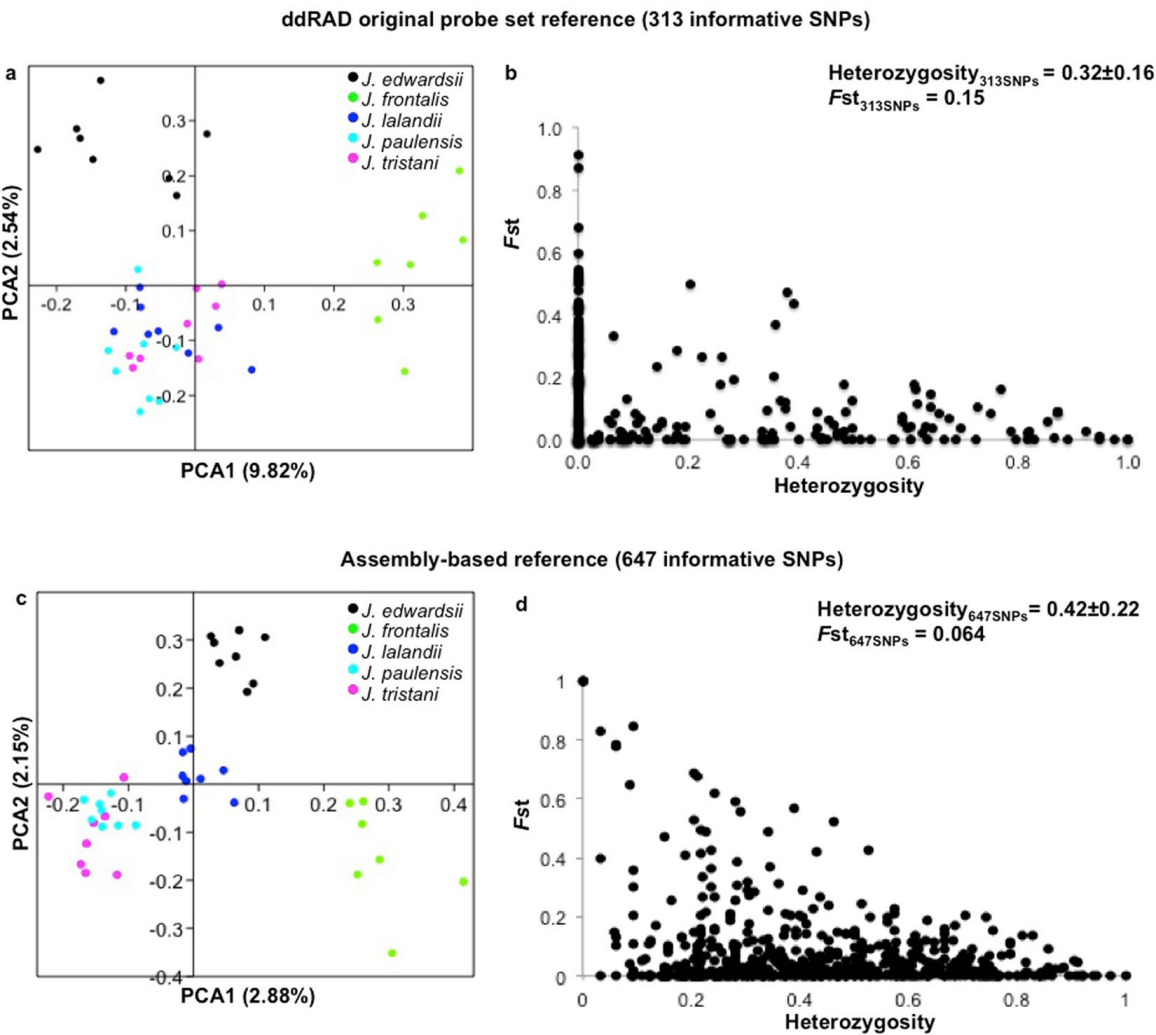
Principal Component Analysis and scatter plot of locus-specific *F*st vs observed heterozygosity of *Jasus* species in modern samples. (**a**) and (**b**) 313 SNPs from the ddRAD original probe set; (**c**) and (**d**) 647 SNPs from the assembly-based reference. Maximum missing data per site was set to 0.10, MAF < 0.05, and overall *F*st was estimated according to Weir & Cockerham (1984).

The variant calling followed rigorous post processing filters to reduce the number of false positive calls, resulting in 5,486 SNPs detected within and among species (Supplementary Table S3). After discarding SNPs in Linkage Disequilibrium, those with greater than 10% missing data or a minor allele frequency (MAF) lower than 0.05, 313 informative SNPs remained across all modern *Jasus* samples. These SNPs had a mean heterozygosity of 0.32 ± 0.16 and a global *F*st^24^ of 0.15. PCA analyses of this data revealed three main clusters (Fig. 3a): (1) *J. edwardsii*, (2) *J. frontalis* and (3) a third cluster comprised by *J. lalandii*, *J. paulensis* and *J. tristani*. Several SNPs (133) were fixed within species (Fig. 3a and b), which is unsurprising given that these SNPs are testing species level differences, however, the remaining SNPs allow population-level assessment within multiple taxa.

### Assembly-based reference for *Jasus* genus based on target enriched sequencing data

In order to enable more effective mapping results across the 40 *Jasus* specimens described above, a new reference sequence was built based on a *de novo* assembly, to account for the entire sequence diversity across non-target *Jasus* species, including ‘off-target’ loci (i.e. flanking regions adjacent to the targeted ddRAD loci). All *Jasus* reads were clustered under a 90% similarity threshold, and the clusters were screened for putative paralogous loci, over-splitting loci, mitochondrial DNA copies and assembly artefacts, such as external contaminants and chimeras, each of which were discarded (detailed in Supplementary Methods S4). The new assembly-based reference comprised 5,940 loci, of which 1,773 loci were assigned as off-target reads, rather than artefacts (Fig. 3b), and therefore, an alternative source of informative sequencing data. BLAST analysis of off-target loci showed 925 significant hits to a *J. edwardsii* transcriptome draft (SRA Bioproject accession number: PRJNA386609), indicating that almost half off-target loci are in fact expressed regions of *J. edwardsii* genome (Supplementary Table S5).

### Mapping efficiency of assembly-based reference in modern samples

The assembly-based reference increased mapping success from 34.86% to 49.23% across the 40 modern *Jasus* specimens (Table 3). Mapping results showed that 1,475 (among 5,940) loci were monomorphic among specimens. Among the variable loci, we found 1,731 homologous loci were consistently shared across the five species, 4,026 were shared across at least three species and 112 loci were found in only a single species (Fig. 2a). The number of loci in each species ranged from 4,106 in *J. edwardsii* to 2,968 in *J. frontalis* (Table 4). Employing an assembly-based reference maximised the use of available reads because it permitted the mapping of loci that were too divergent - in the non-targtet species - to be efficiently mapped to the original probe set.

**Table 4:** Descriptive statistics of mapped data based on the new assembly for modern and museum samples.

*Species*	Date	N	Mapping results				Gene diversity							
			Overall coverage	Mean coverage among samples	Read heterozygous rate	% GC	Loci counts	Variable loci	Deamination	SNPs	SNP/locus	tsi/tsv	MMAF	HHet.
**Modern samples**														
*J. edwardsii*	2013	8	48.89 ± 1.46	8.39 ± 0.76^a^	0.0066 ± 0.0006	44.96 ± 0.88^a^	4,106	3,363	0.861	30,334	9.02	1.28	0.28	0.27
*J. frontalis*	2010	8	11.25 ± 0.36	3.21 ± 0.14^b^	0.0271 ± 0.0035	47.32 ± 0.31^b^	2,698	1,375	0.897	6,632	4.82	1.24	0.32	0.21
*J. lalandii*	2015	8	20.69 ± 0.49	4.02 ± 0.26^a^	0.0151 ± 0.0015	43.41 ± 0.10^a^	3,297	2,338	0.883	17,223	7.37	1.31	0.28	0.2
*J. paulensis*	2015	8	40.56 ± 1.16	7.00 ± 0.33^a^	0.0082 ± 0.0005	45.00 ± 0.14^a^	4,035	3,261	0.86	28,673	8.79	1.28	0.28	0.24
*J. tristani*	2015	8	18.50 ± 0.53	3.90 ± 0.42^c^	0.0152 ± 0.0036	45.28 ± 0.45^a^	3,735	2,604	0.888	16,121	6.19	1.28	0.3	0.21
**Museum samples**														
*J. caveorum*	1995	7	30.28X ± 0.94	7.01 ± 1.74	0.0090 ± 0.0020	44.29 ± 1.00	3,828	2,745	0.864	18,140	6.61	1.27	0.33	0.37
*J. edwardsii*	1991	8	17.63X ± 0.48	3.55 ± 1.00	0.0170 ± 0.0070	43.68 ± 1.10	3,377	2,103	0.887	12,782	6.08	1.3	0.41	0.35
*J. lalandii*	1967	8	14.15X ± 0.74	9.38 ± 1.97	0.0136 ± 0.0045	43.00 ± 1.48	2,248	982	0.936	4,408	4.49	1.22	0.34	0.28
*J. lalandii*	1991	8	35.19X ± 1.93	4.02 ± 1.46	0.0103 ± 0.0025	37.05 ± 0.64	1,043	315	0.835	941	2.99	1.1	0.4	0.55
*J. paulensis*	1967	8	5.96X ± 0.22	2.38 ± 0.17	0.0170 ± 0.0031	36.66 ± 0.37	1,380	483	1.00609	1,978	4.1	1.19	0.36	0.19

Mean coverage and GC content (GC%) per sample was compared across species in modern samples. Data expressed as mean (SEM). ^a,b,c^Different superscripts within a column denote significant differences (P < 0.01). Adjustment for multiple comparisons: Bonferroni.

### Genome divergence of non-target *Jasus* species

Examination of target enriched sequencing efficiency revealed evidence of genome divergence among *Jasus* species. Aspects such as sequencing yield (given in number of processed reads), mean coverage, GC content (%GC) and SNP diversity suggest important differences across the captured genomes (Table 4). For example, the heterogeneous mean coverage (0.631; P < 0.01) and GC content (0.346; P < 0.01) across species, for example, were both positively correlated with sequencing yield (Supplementary Table S6). However, mean coverage and GC content were not reciprocally correlated. The mean coverage was significantly reduced in *J. tristani* and *J. frontalis* (3.90X ± 0.42 and 3.21X ± 0.14 respectively); while the former presented the lowest sequencing yield (Table 3), the latter presented the greatest GC content. This implies that, unlike *J. tristani*, *J. frontalis* might have deeper genome divergence from the other *Jasus* species (as suggested by the PCA analyses in figure 3). This may have also affected the mapping efficiency in this species.

#### Sequencing coverage

The overall mapping coverage (mean depth of targets) of the assembly-based reference varied from 11X in *J. frontalis* to 48X in *J. edwardsii* (Table 4). In terms of sequence similarity, this means that the efficiency of probes in capturing the genome targets was reduced in *J. frontalis* and increased in *J. edwardsii*. *J. edwardsii* showed the best results in terms of captured yield and coverage (Tables 3 and 4) as sequences of this species were used to design probes (along with *S. verreauxi*). On the contrary, the *J. frontalis* genome was the most divergent as evidenced by higher sequence dissimilarities to the assembly-based reference. In line with this observation, differences in average sequence coverage have been reported in target-capture experiments for species with more than 5% sequence divergence^25–28^. However, *J. frontalis* did not have the lowest sequencing depth or captured yield as expected, but rather the lowest number of mapped reads (Table 3) and also the lowest mean target coverage (Table 4). Although this could suggest differentiation bias as a result of low sequence coverage, such bias tends to underestimate rather than overestimate differentiation and rare variants^29^.

#### SNP diversity

Overall 23,555 cross-species variants were identified within the 1,731 shared loci (Fig. 2a) from the assembly-based reference. The diversity of this reference is in accordance with the patterns in the original mapped loci (Table 4), with the lowest number of variants within species found in *J. frontalis* 2,698 and the greatest in *J. edwardsii* (4,106). After removing SNPs with missing data greater than 10%, MAF lower than 0.05 and linked SNPs, a total of 647 informative SNPs remained (2X increase from the original mapped loci). This SNP subset displayed a high proportion of polymorphic loci (0.42 ± 0.22 mean heterozygosity) and low differentiation level (global *F*st = 0.064) among species (Fig. 3d). This indicates that they are potentially more informative within species rather than among species comparisons, when compared with those from the mapped probe set. For example, in Fig. 3d, several loci (232 SNPs) present *F*st among species equal to 0. The PCA analyses based on 647 SNPs revealed four main clusters: (1) *J. frontalis*, (2) *J. edwardsii*, (3) *J. lalandii* and (4) a cluster comprised by *J. paulensis* and *J. tristani* (supporting Groenenveld *et al*. (2012)^30^ findings, based on mtDNA data evidence, that *J. paulensis* and *J. tristani* should be synonymized). The assembly-based data set provided greater separation than the mapped probes, placing *J. lalandii* in a different cluster from the *J. paulensis*/*J. tristani* species. This result indicates that differentiation levels among non-target species can be more accurately achieved by building an assembly-based reference of target-enriched sequencing data; because it takes into account the full diversity of the sequencing dataset.

### Efficiency of target enriched sequencing method applied to museum samples

In order to evaluate the efficiency of target enriched sequencing in museum samples, reads from the 39 museum specimens of five *Jasus* species (Table 4) were mapped to the assembly-based reference. The alignments were then compared with modern samples in terms of mean coverage, GC content, read heterozygous rate and SNP diversity. Prior to this comparison, DNA damage patterns were established for each museum sample by tracking and quantifying cumulative substitutions frequencies of C to T at the 5′end and G to A at the 3′end in mapped reads using the mapDamage 2.0 software^31^ (data not shown). No evidence of base mis-incorporation bias due to DNA damage was found in any of the museum samples.

#### Sequencing coverage and GC content

**Figure 5:**
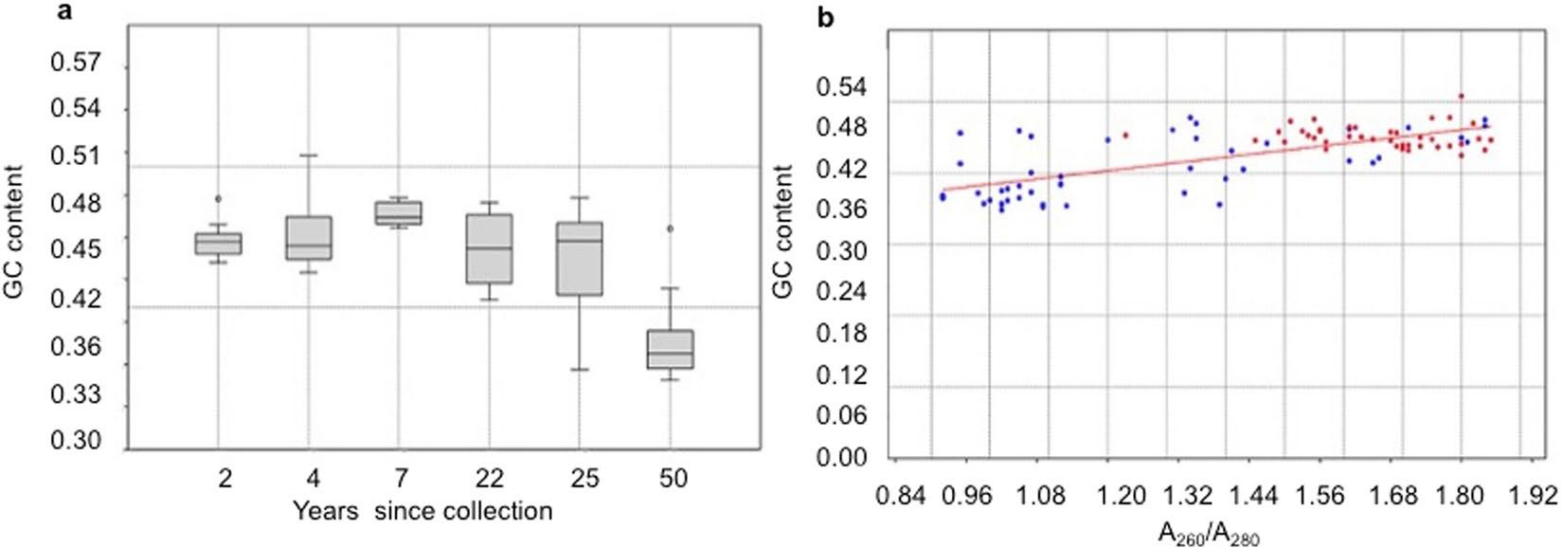
Sample quality of modern and museum *Jasus* specimens. (**a**) Box-plot of mean GC content and year since sample collection. (**b**) Linear regression between A_260_/A_280_ ratio and GC content; blue dots represent museum samples and red dots represent modern samples.

In contrast to the modern samples, a significant correlation between GC content and mean coverage (0.24179; P < 0.032) was found, suggesting that museum samples were highly impacted by sequencing coverage (Supplementary Table S7 and Fig. S8). The overall GC content among museum samples were quite heterogeneous (Fig. 5a) and significantly correlated to the year of collection (0.483; P < 0.01) and A_260_/A_280_ ratio (0.482; P < 0.01) (Table S7 and Fig. S8). We suggest that sequencing coverage was affected by DNA fragmentation in museum samples leading to non-uniform representation of targets and heterogeneous GC content in sequencing libraries. However, patterns are difficult to interpret. For example, the highest overall coverage was observed in 50-year-old *J. lalandii* samples (35.19X ± 1.93) and the lowest in 50-year-old *J. paulensis* (5.96X ± 0.22) (Table 3). In both sample groups, the mean GC content significantly deviated from the mean of corresponding modern samples (P < 0.01 in both cases; Supplementary Table S9), suggesting significant bias among these samples. However *J. paulensis* museum samples were the only samples that demonstrated an increased read heterozygous rate (P < 0.0066; in Supplementary Table S9). The impact of sample age in DNA fragmentation was evident, but not homogeneous, among museum samples due to variability in DNA purity as indicated by the A_260_/_A280_ ratio as shown in Fig. 5b. It may also be related to the museum preservation methods applied to the specimens.

#### Utility of museum samples

In an attempt to find an approach to screen the museum samples for library preparation in future studies where there is likely to be DNA damage and fragmentation, we found that samples with A_260_/A_280_ ratios as low as 1.01 and moderate DNA concentration above 30–40 ng/µL (as determined from the A_260_ values) exhibited acceptable average target coverage with no significant GC% deviations (Fig. 5b). This allows false-positive variants to be filtered, improving SNP calling accuracy^32^, and, a ‘rule of thumb’ for selecting suitable historic samples for library preparation; samples with an A_260_/A_280_ ratio greater than 1.0 and spectrophotometric quantification greater than 30 ng/µL are more likely to generate acceptable sequencing coverage. The A_260_/A_280_ ratio is routinely used as a DNA purity indicator for protein, phenol or other contaminants that strongly absorb light at or near 280 nm wavelengths, however, the actual ratio is also subject to nucleotide composition and pH variation^33^. Therefore, when using historic samples for library preparation, close attention should be paid to both the A_260_/A_280_ ratio and DNA spectrophotometric quantification cutoff, both prior to sequencing, and when assessing the results of sequencing.

### Mapping efficiency of assembly-based reference in museum samples

Because of the limitations of the museum samples used in this study, the amount of missing data was much higher in these samples. This is likely because the fragmentation bias of DNA templates in library preparation led to uneven enrichment and sequencing coverage of targeted loci. The number of loci in each species ranged from 3,828 in 25-year-old *J. edwardsii* to 1,043 in 50-year-old *J. lalandii* (Table 4). Each of the four species for which museum samples were used, has at least one fold fewer loci (nearly five fold in *J. paulensis*) when compared with the modern samples. The loci also seem to be less variable, as determined by the number of SNP per locus in all species. Whilst cumulative substitution bias was not evident in museum samples, the overall deamination level in museum samples of *J. lalandii* and *J. paulensis* from the year 1967 was higher than modern samples (Table 4). Thus, in order to examine the usefulness of the target capture approach for incorporating museum samples into population genomic studies, we modified the variant-filtering settings to avoid false positive variants as a precaution. Strand biased variants, heterozygous SNPs at the end of reads and SNPs with deamination pattern found towards the 5′ and the 3′ reads’ ends were filtered out resulting in a slightly different SNP set from that used for the modern samples.

### Genetic diversity of *Jasus* including museum samples

**Figure 4:**
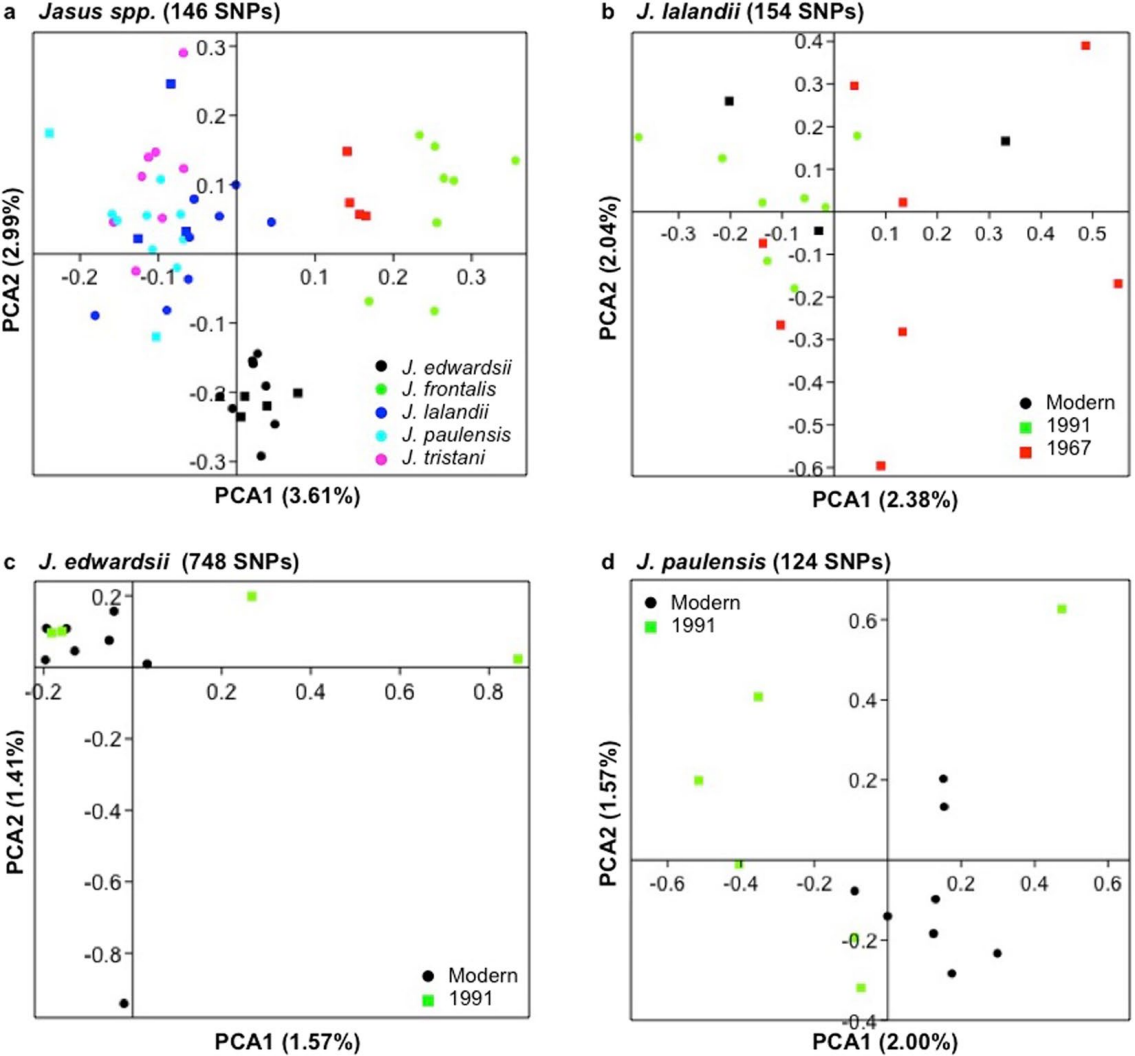
PCA across and within species based on SNP genotypes, where no deamination filter was applied. For each dataset, the sample passed filters and SNP pruning was adjusted to allow 0.10 maximum missing data per site and MAF < 0.05: (**a**) PCA across six *Jasus* species, 146 SNPs and 53 samples passed filters; (**b**) PCA of *J. lalandii*, 154 SNPs and 19 samples passed filter; (**c**) PCA of *J. edwardsii*, 748 variants and 12 samples passed filters; (**d**) PCA of *J. paulensis*, 124 variants and 14 samples passed filters.

Amongst the *Jasus* species, only museum samples were available for *J. caveorum*. This species formed a distinct group in the PCA (Fig. 4a). However, the necessity of using the reduced set of SNPs meant that again, *J. lalandii* appeared indistinguishable from *J. paulensis*/*J. tristani*. Filtering SNPs for deamination had a negligible impact on the overall results, as evident from global *F*st estimates (from 0.0589 to 0.0653 after filtering) and PCA distributions (Supplementary Fig. S10). At the intraspecific level, deamination filtering also had little impact on *F*st values, suggesting that base modifications due to DNA damage are not severe enough to influence overall differentiation or are not present among the variants called. Thus, provided historic samples with extreme low coverage (<2.0X) are removed, variant call accuracy can be adjusted to diminish base mis-incorporation bias to a negligible level.

#### Museum samples versus modern samples

PCA plots of modern versus museum samples demonstrate that there is a gradient of variation related to the first principal component (PC1) for both *J. lalandii* (Fig. 4b) and *J. paulensis* (Fig. 4d), but not for *J. edwardsii* (Fig. 4c). Given that the aims of a museum/modern sample comparison are to assess temporal changes in population structure, these results are clearly interesting and should be investigated further using population-sequencing data. Our results demonstrate that target capture of ddRAD loci of lobster specimens from museum collections will likely provide usable sequencing data for population genomic approaches, but that an increase in sequencing effort of historical samples might be necessary to enable confident variant calling. Ideally, the coverage in historical samples should be similar to modern specimens, however DNA fragmentation or low DNA quantity will negatively impact the sequencing depth; in some cases the increase of sequencing effort might not affect the coverage of targets in these samples. This caveat requires further investigation in future studies by comparing population genetic parameters such as *F*st, demography inference, selection^34^ and the scaled population mutation rate θ^35^.

## Conclusions

### Keep it simple and effective

Here, ddRAD libraries from two closely related lobster species, *J. edwardsii* and *S. verreauxi* were used as genome resources to design probes to capture and enrich genomic libraries of other five closely related species. The target-enriched sequencing generated thousands of informative markers for population genomics application with a small sequencing effort of 4.6 Gbp only. The enriched sequencing of previously discovered ddRAD loci enabled the recovery of 1,250 out of 2,366 ddRAD loci in the species from which the probes were designed, including sample replicates between both methods. Thus, it could potentially be used to enrich and re-sequence ddRAD loci outliers discovered in previous studies^15^ in a range of populations to investigate genome signatures of selection. Also, this method circumvents one of the main disadvantages of ddRADseq, that being the high level of missing data due to allele dropout.

Our target-enriched loci were consistently recovered across all non-target *Jasus* species, whilst the use of an assembly-based reference including off-target sequences substantially increased mapping efficiency and average target coverage of informative loci. This enabled the detection of 1,731 loci shared across five *Jasus* species, which has the potential to enable direct comparison of locus-specific population genetic variation (including putatively selected loci) over multiple species, something that is not possible using restriction enzyme-based approaches. Thus, we provided a robust method approach to interrogate comparative studies of dispersal, self-recruitment and adaptation in all *Jasus* populations. Given the limited number of genetic markers available for wide comparisons of *Jasus* species distributions, the ddRAD loci enriched data represents a reliable genome resource, highly repeatable among individuals and replicates. This represents a permanent resource that may be further used for comparison among different studies in *Jasus* species, and perhaps to other closely related species, such as *S. verreauxi* or *Projasus* spp.

We have also developed a suitable protocol for the use of museum samples in population genomics studies. These samples provide representation of temporal shifts in populations, and critically are sometimes the only available material for a species. Based on the present results, informative SNPs from museum samples could be obtained provided a minimum target sequencing coverage exists and reads are not subject to base mis-incorporations bias. Here, we compiled a series of protocols for ddRAD loci enriched sequencing that enables quality control of sequencing data by computationally removing contaminants, PCR duplicates and spurious variants due to base mis-incorporation. This is particularly relevant when dealing with museum samples that are often contaminated, present low DNA yield and severe DNA damage. Thus, the methods described here can be applied to further investigate temporal changes in population structure, a critical issue in intensively fished species^36^.

## Methods

### Target species

The genus *Jasus* encompasses six lobster species (*J. caveorum*, *J. edwardsii*, *J. frontalis*, *J. lalandii*, *J. paulensis* and *J*. tristani) that are distributed throughout the Southern Hemisphere^37^. *J. edwardsii* is the most widespread species within the genus, whilst the other species maintain limited geographical distributions, with some species known from only a single seamount (e.g. *J. caveorum*). These species all support valuable fisheries, and have been exploited for more than one hundred years^38^.

Due to their markedly long pelagic larval durations (PLD; in the order of 18–24 months^39^), panmixia (i.e., random mating between all individuals) is still widely accepted for some spiny lobster species, especially in the context of fishery management resources^33^. However, the paradigm that marine species with pelagic larval stages are genetically homogeneous across large geographic scales^40^ is changing and cannot be systematically extrapolated across species. For instance, despite the high dispersal potential of *J. edwardsii* due to its long PLD, the occurrence of panmixia between Australian and New Zealand populations has been rejected^15, 41, 42^. Given the importance of understanding gene flow for fisheries management, these species represent an ideal case for the utilisation of genomic resources to better understand population structure. Currently, for species other than *J. edwardsii*, the genetic markers available are limited to mtDNA^30^, which is inadequate for accurately assessing population structure.

### Sample collection

For the target-capture experiment, we collected 87 samples of modern and museum specimens of *Jasus* comprising six species *J. edwardsii*, *J. caveorum*, *J. frontalis*, *J. lalandii*, *J. paulensis* and *J. tristani*. Specimens of *S. verreauxi* (N = 16) were included to evaluate the custom probe set and the efficiency between ddRAD and target-capture methods for the validated loci in both methods (Supplementary Table S11).

#### Modern samples

A total of 40 lobsters samples were collected between 2010 and 2015. The French Southern and Antarctic Lands (Terres Australes et Antarctiques Françaises-TAAF) provided *J. paulensis* pleopod tissue collected in 2014. *J. lalandii* and *J. tristani* pereiopod samples, collected during in 2015 in South Africa and Tristan da Cunha islands, respectively, were donated by the South African Department of Agriculture, Fisheries and Forestry. *J. frontalis* were sampled in Juan Fernandez archipelago in 2010^43^, these samples were donated by the Universidad de Concepcion (Chile).

#### Museum samples

Museum samples (N = 47) were donated by the National Institute of Water and Atmospheric Research (NIWA) and Te Papa Museum (New Zealand). The samples were collected in 1967 (*J. lalandii* and *J. paulensis*), 1991 (*J. edwardsii* and *J. lalandii*) and 1995 (*J. caveorum*). Samples were preserved in ethanol/isopropanol (mostly evaporated). In some cases preservation methods were not indicated.

### Loci selection from ddRAD libraries for probe design

We used the ddRAD libraries published by Villacorta-Rath *et al*.^15^ for *J. edwardsii*, and produced a subsequent library for the closely related *S. verreauxi* for our probe design. These species have estimated divergence time of approximately 40^44^ to 108^45^ million years. A modified Peterson *et al*.^46^ ddRAD protocol was utilized for library preparation and sequencing of both *J. edwardsii* and *S. verreauxi* DNA samples, as described in Villacorta-Rath *et al*.^15^. Full information of samples from which ddRAD libraries were used for probe set design are described in Supplementary Table S12. The ddRADseq indexed libraries of 42 *J. edwardsii* and 55 *S. verreauxi* individuals were sequenced on the Illumina MiSeq platform. Raw ddRAD reads were trimmed using Trimmomatic 0.32^47^, in order to remove poor quality sections or removed the entire read when the average Phred score was lower than 33. Contaminant reads were identified using Kraken 0.10.4 beta^48^ and subsequently removed. Sequences were paired and trimmed to a minimum size of 140 base pairs (bp) using Pear 0.9.4^49^. Paired reads were then demultiplexed and assigned to corresponding samples following the dual indexed adapter sequences by using a locally developed pipeline (https://github.com/molecularbiodiversity/rad-pipeline).

#### PyRAD assembly and ddRAD loci catalogue

Remaining ddRAD reads were assembled in PyRAD 3.0.4^19^ and used to build ddRAD loci catalogues within and between species. Samples were assembled in three datasets as follows: (1) *J. edwardsii* only; (2) *S. verreauxi* only; and (3) all samples from both species. For assemblies 1 and 2 we adopted a 95% similarity threshold within species and for assembly 3 we established an 85% similarity threshold. A maximum of three mismatches and a maximum of 0.5 site heterozygosity were allowed per cluster. To avoid clusters of paralogous loci we discarded all clusters with excessive shared heterozygous SNPs in more than four individuals and the paralogous filter was set to three. The ddRAD loci were considered for probe design (candidate ddRAD loci) only if they were shared across 10 individuals in assemblies 1 and 2 or 20 individuals in assembly 3. PyRAD assemblies’ settings and outputs are detailed in the Supplementary Methods S1.

#### Paralogous loci removal and repeat masking

The candidate ddRAD loci were assembled *de novo* adopting a 75% similarity threshold using Geneious R7^50^. The default settings of RepeatExplorer^51^ were implemented to distinguish paralogous families^52^, and contigs with more than 90% similarity across at least 55% of the fragment length were discarded. Highly similar clusters with spurious alignments resulting from low complexity DNA sequences (comprised by mononucleotide repeats) were also discarded. As capture probes are known to support up to about 12% sequence divergence^13, 53^, 75% similarity among ddRAD loci was used as a limit. Remaining repetitive regions among ddRAD loci were identified and removed using RepeatMasker Web server^54^, with *Homarus americanus* as model organism, whilst the ddRAD loci derived from mitochondrial genome were identified using the *S. verreauxi* mitochondrial DNA complete genome (AB859775 accession number)^55^.

#### MYbaits® probes panel

A total of 2,366 loci were finally selected as templates for bait manufacturing: 2,358 nuclear loci and eight mitochondrial loci. The MYbaits^®^ set were estimated to cover 322 kb from 5.3 Gb of *J. edwardsii* genome, including 80 loci identified as outlier SNPs putatively under selection in *J. edwardsii* ^15^. In total, 4,732 120-mer MYbaits^®^ probes (MYcroarray) were manufactured with 2X tiling density and an overlap of 60 bp between probes. Probes sequences were deposited at the Dryad data repository (http://dx.doi.org/10.5061/dryad.3dk40).

### Target enriched sequencing

#### DNA extraction

DNA was extracted from adult lobsters (pleopod clip or pereiopod muscle) and phyllosoma larvae (leg). All DNA extractions were performed with the DNeasy Qiagen kit using spin columns EconoSpin (Epoch Life Sciences). As the museum specimens resulted in low DNA yield and purity levels, the isolation protocol was optimized accordingly. For these samples, DNA recovery was improved by overnight incubation, final DNA elution in 30 µl of AE buffer with three consecutive washes followed by column centrifugation. DNA extraction and library preparation using museum specimens was performed using consumables dedicated to the museum specimens only with different batches for each species.

### Library preparation

Genomic DNA samples from modern specimens were randomly fragmented by sonication using a BioRuptor NGS (Diagenode). Most museum DNA samples were naturally fragmented (i.e. due to degradation over time) therefore a fragmentation step was not necessary. We followed an established dual-indexed library preparation protocol detailed in Rohland and Reich^56^ for hybridization capture reaction, incorporating few modifications to reduce costs and hands-on time. We omitted the size-selection, as it is assumed that sonication shearing generates a sufficiently narrow fragment-size distribution^57^ and re-dimensioned final volumes of reactions and the proportion of magnetic microspheres (hereafter referred to as magnetic beads) within the purification steps (detailed in Supplementary in Table S13). Libraries were purified with magnetic beads, eluted and multiplexed by pooling eight of 16 libraries, prior to hybridization capture reaction^58^ at equimolar ratios to a final yield of 200–500 ng (Supplementary Table S14 and S15). Two negative controls were included in every step.

### Target-capture

The MYbaits^®^ manufacturer’s protocol was followed, replacing the blocker #3 of the kit with the custom blocking oligonucleotides described in Rohland and Reich^54^. In-solution hybridization reactions of libraries with biotinylated probes were incubated for 24 hours at 65 °C. Hybridized fragments were captured with Streptavidin-coated magnetic beads and washed four times with Wash Buffer (MYbaits^®^ kit component) for five minutes to remove unspecific material. Captured DNA fragments were denatured for five minutes at 95 °C and eluted in TE buffer. The enriched multiplexed libraries were PCR-amplified for 18 cycles (Supplementary Table S15). Prior to sequencing, enriched libraries were Qubit-quantified, normalized and quantified in real-time PCR (Supplementary Table S14). Cluster generation was performed in a single Illumina MiSeq. 1500 lane, with 2 × 250 cycles of base incorporation.

### Sequencing data analysis

#### Sequencing data processing

Sequencing data was processed using the locally developed pipeline CarlaSeq (https://github.com/molecularbiodiversity/carlaseq) described in detail in Supplementary Methods S4. Briefly, it involved the first four steps of the pipeline that consisted of adapter trimming, removal of contaminants (human and microorganisms), merging of paired-end reads and de-multiplexing. The paired-end filtered reads were merged and trimmed to fragments up to 220 bp long. Reads with average Phred score lower than 33 were discarded.

#### Read mapping

Processed reads were mapped to the reference ddRAD loci, using Bowtie 2^21^ with ‘–very-fast-local’settings. SAMtools (http://github.com/samtools/samtools) was used to sort alignments (maQ ≥ 5). Picard 2.6.0 (http://broadinstitute.github.io/picard) was used to mark PCR duplicates from alignments by identifying fragments that are identical in insert length and related sequence composition. The capture reaction ensures that single strand fragments at a given locus are unlikely to be of equal length unless they are duplicates.

#### Variant-calling

Because most population genetic models are vulnerable to NGS errors (sequencing errors) and the amplification of PCR duplicates, initial variant-calling was only performed in modern samples to estimate overall genetic variation across species obtained with our capture probe set. Museum samples were initially omitted in order to reduce the chance of detecting false positive variants resulting from incorporated errors and DNA damage causing base modification. Thus, modern sample alignments were merged and post processing filters were used to perform probabilistic variant-calling using GATK 3.6^59^. GATK best practice guidelines were adapted as follows: first, indel intervals were locally realigned and the variants detected were indexed and converted into a Variant Call Format (VCF); second, variant-filtering was set to stringent thresholds for strand bias, coverage, mapping quality and variant position. Specifically, SNP sites with quality by depth <4, root mean square mapping quality over all the reads at the site <18, Phred quality <40, Fisher Strand >60, haplotype score >13 and less than 5X coverage across at least eight individuals were discarded. Heterozygous SNPs at read ends (reads rank position <−12bp) were also discarded. Only biallelic sites that passed all filters were retained so that data were directly comparable among all *Jasus* species.

Deamination and transition/transversion ratio were calculated over filtered variants using VCFtools 0.1.14^60^. SNP pruning, diversity indices (*F*st, heterozygosity and MAF) and PCA were estimated for all *Jasus* species using Plink 1.9^61^.

### Method efficiency

The efficiency of the target capture experiment was firstly assessed for the two species (*J. edwardsii* and *S. verreauxi*) from which the original probes were designed. This data set included 11 *J. edwardsii* specimens and 16 *S. verreauxi*, of which eight and 16 samples, respectively, were replicates of the original ddRAD libraries sequenced. The number of on-target reads between species was compared using BLAST. Then, mapping success (Bowtie 2) and the amount of missing data (loci counts) per number of samples were compared between ddRAD and target-capture methods for both species.

The efficiency of the target capture experiment non-target *Jasus* species was assessed using only modern samples (N = 40). The overall target coverage was given by the mean depth of targets for each species using BEDtools 2.26^62^. Mean coverage (sample coverage depth), heterozygous read rate and GC content of samples were estimated from sample alignments using BBmap (https://github.com/BioInfoTools/BBMap).

In order to compare the target-genome divergence among species and its effects on target enriched sequencing, a nonparametric correlation was performed among sequencing yield (given in number of processed reads), mapped reads, GC content, mean coverage and mapping quality for each species using SPSS v22.0 (IBM Corp., NY, USA). Univariate analysis of variance was applied to test whether GC content and mean coverage were significantly different among *Jasus* species. The sequencing yield effect was included as a covariate, since it was previously explored as a random factor within a one-way ANOVA analysis and found to be significant. Data were checked with Levene’s test and logarithm transformed to ensure normality and homogeneity of variance. Principal Component Analysis (PCA) including all variables was performed using PAST 3.12 (http://folk.uio.no/ohammer/past/).

### Assembly-based reference building

Clustered assembly using Vsearch 1.1.3 (https://github.com/torognes/vsearch) was performed only on modern samples in order to build a reference loci catalogue avoiding low quality reads and potential artefacts from museum samples. The similarity threshold was set to 90% to account for sequence diversity within and among *Jasus* species. Resulting clusters with less than 40 reads were discarded. To ensure that clusters correspond to different genomic regions a *de novo* assembly was performed using Geneious R7^48^ to check overlapping (redundancy) between clusters. The Geneious assembler was set to custom sensitivity parameters of 120 bp minimum overlap among clusters, which corresponds to the capture probe length, and 85% similarity threshold. Clusters assembled into the same contig were catalogued as a single locus. A reciprocal BLAST^20^ using the original probe set database was applied to identify and filter only on-target clusters. These steps are detailed in CarlaSeq (https://github.com/molecularbiodiversity/carlaseq) pipeline in Supplementary Methods S4 (see steps five to eight).

### Assessing diversity using museum samples

Evidence of DNA damage in museum samples was established by tracking and quantifying cumulative substitutions frequencies of C to T at the 5′end and G to A at the 3′end in mapped reads using mapDamage 2.0^31^ (https://github.com/ginolhac/mapDamage). We also estimated the deviations of GC content and target genome heterozygous rate with BBmap (https://github.com/BioInfoTools/BBMap) between modern and museum samples as a result of low coverage. A Student’s t-test (SPSS v22.0, IBM Corp., NY, USA) was used to test whether GC content and heterozygous rate between modern and museum samples for each species were significantly different. Data were checked with Levene’s test, and degrees of freedom were adjusted using the WelchSatterthwaite method for data groups with unequal variances. Results are expressed as means ‘ ± ’ standard errors (SEM), with statistically significant differences stated at P-value < 0.01. In order to evaluate the effect of DNA purity on target enriched sequencing among museum samples, nonparametric correlation analyses were performed among sequencing yield, mapped reads, mean coverage, mapping quality, GC content, year of collection and A_260_/A_280_ ratio including modern samples (N = 79). PCA including all variables was performed using PAST 3.12 (http://folk.uio.no/ohammer/past/).

According to Parks *et al*.^32^ variant-calling is not significantly affected by low damage (base modifications) in DNA, but is highly impacted by low coverage depth at any level of divergence. Thus, to avoid introducing severe bias by extreme low coverage depth, all samples with less than 2.0X target coverage (mean depth among targets) were removed from population genetics analysis as follows: three *J. caveorum* individuals, three *J. edwardsii* individuals, five *J. lalandii* from 1991 and two *J. paulensis* individuals from 1967. Note that Bi *et al*.^2^ previously adopted a similar threshold for museum samples of non-model species, when utilizing a probabilistic method to call variants. *J. paulensis*, *J. lalandii* and *J. edwardsii* remaining samples collected in different decades were used to make comparisons between modern and museum samples based on unbiased *F*st^24^ values and PCA. As a proof-of-concept of the negligible DNA damage of museum samples used in this study, deamination filters were applied to perform a before/after comparison as recommended in Bi *et al*.^2^. Data was cleaned up for G to A and C to T substitution sites detected in historic samples. This is due to the fact that deamination substitutions are directional, they substitute G to A and C to T, but not A to G or T to C. Major and minor alleles were detected for all loci and directional substitutions G to A and C to T intervals were removed from all samples (modern and museum).

## Electronic supplementary material

Supplementary information
