# Supplementary material for: Efficiency of ddRAD target enriched sequencing across spiny rock lobster species (Palinuridae: Jasus)
Source: Sci Rep. 2017 Jul 28;7:6781. doi: 10.1038/s41598-017-06582-5 (PMC5533801; doi:10.1038/s41598-017-06582-5)
Supplement: Supplementary file 1 — Supplementary information [file 41598_2017_6582_MOESM1_ESM.doc]

**Efficiency of ddRAD target enriched sequencing across spiny rock lobster species (Palinuridae: *Jasus*)**

# Carla dos Anjos de Souza^1,*^; Nicholas Murphy^1^; Cecilia Villacorta-Rath^2^; Laura N. Woodings^1^; Irina Ilyushkina^3^; Cristián E. Hernández^4^, Bridget S. Green^2^; James Bell^3^, Jan M. Strugnell^5,1^

^1^Department of Ecology, Environment & Evolution, School of Life Sciences, La Trobe University, Melbourne, VIC 3086, Australia

^2^Institute for Marine and Antarctic Studies, University of Tasmania, Hobart, TAS 7001, Australia

^3^School of Biological Sciences, Victoria University of Wellington, Wellington 6140, New Zealand

^4^Departamento de Zoología, Facultad de Ciencias Naturales y Oceanográficas, Universidad de Concepción, Concepción, Chile

^5^Centre for Sustainable Tropical Fisheries and Aquaculture and College of Science and Engineering, James Cook University, Townsville, QLD 7001, Australia

*c.dosanjosdesouza@latrobe.edu.au

**Methods S1.** Parameters applied in PyRAD assemblies 1 (*J. edwardsii*), 2 (*S. verreauxi*) and 3 (combined dataset from both species) to generate loci catalogue. N is number of samples; ddRAD loci is the total number of loci resultant from each assembly after redundance/paralogous filtering. Max Het – maximum heterozygosity allowed per samples; Shared Het: maximum number of heterozygous sites among samples.

The PyRAD assemblies resulted in a catalogue of 4,629 loci. *De novo* assembly using more liberal similarity thresholds (75%), revealed a number of possible paralogous loci, which were discarded, and redundant loci among assemblies were synonymized into single loci. 2,390 ddRAD loci (~140 bp length) were retained as potential candidates. These included 746 loci from the assembly 1 (*S. verreauxi* dataset), 468 from the assembly 2 (*J. edwardsii* dataset*)* and 1,176 from the assembly 3 (both species datasets).

The species-specific loci counts overall the three assemblies revealed that 123 loci were shared across the two species. In other words, among the 1,176 loci resultant from the assembly 3, only 123 loci, were in fact found in samples of both species in the dataset.

| **Assembly Dataset** | **Species** | **N** | **Parameters** | | | | **ddRAD loci** |
| --- | --- | --- | --- | --- | --- | --- | --- |
|  |  |  | **Similarity** | **Mismatches** | **Max Het.** | **Shared Het.** |  |
| *1* | *J. edwardsii* | 42 | 95% | 3 | 0.5 | 4 | 468 |
| *2* | *S. verreauxi* | 55 | 95% | 3 | 0.5 | 4 | 746 |
| *3* | *S. verreauxi* and *J. edwardsii* | 97 | 85% | 3 | 0.5 | 4 | 1176 |

**Figure S2.** Trimmed and mapped reads counts per samples.


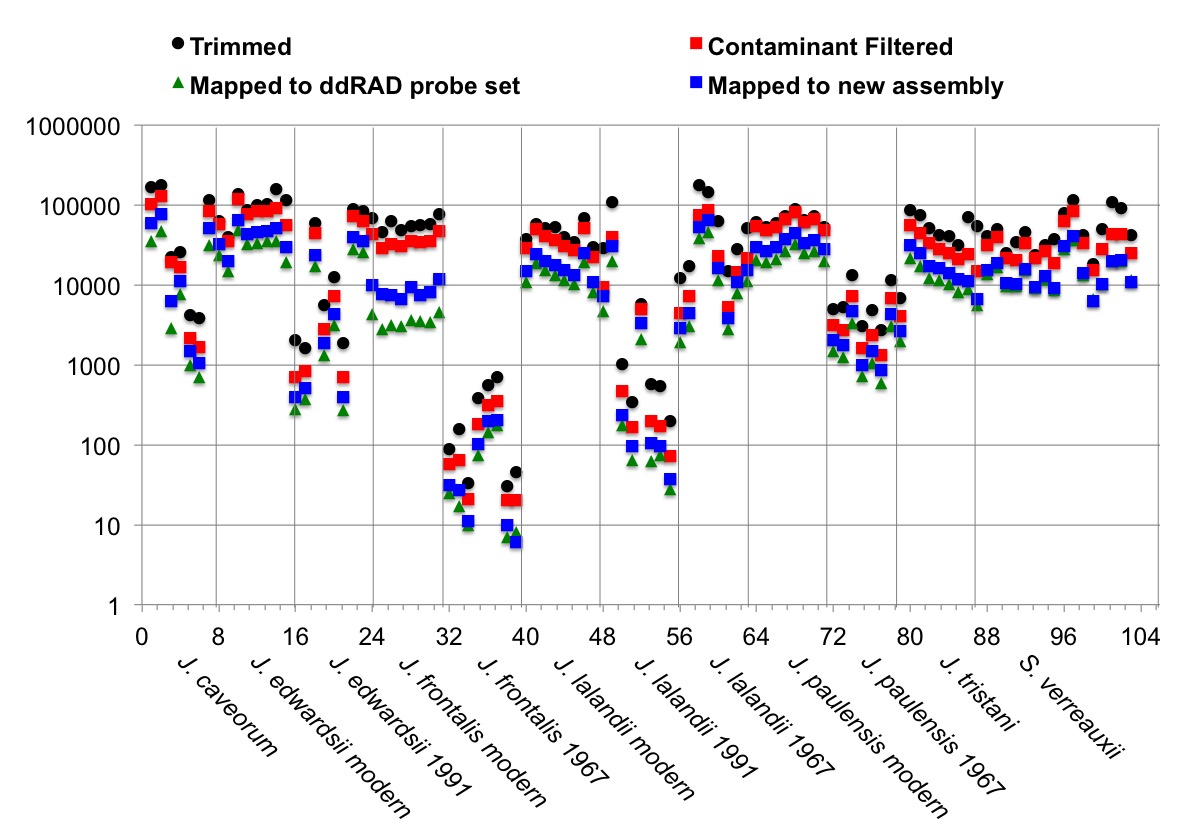


**Table S3.** SNP summary within species in modern dataset based on data mapped to ddRAD loci original probe set. MAF- overall SNP Minor Allele Frequency; Het - overall SNP heterozigosity.

| Species | Year | Loci counts | Variable loci | Deamination | SNPs | SNP/locus | tsi/tsv | MAF | Het |
| --- | --- | --- | --- | --- | --- | --- | --- | --- | --- |
| *J.edwardsii* | 2013 | 910 | 764 | 0.926955 | 6,182 | 8.09 | 1.54 | 0.30 | 0.40 |
| *J.frontalis* | 2010 | 565 | 337 | 0.90832 | 4,003 | 11.88 | 1.62 | 0.32 | 0.29 |
| *J.lalandii* | 2015 | 854 | 632 | 0.930397 | 5,833 | 9.23 | 1.55 | 0.30 | 0.35 |
| *J.paulensis* | 2015 | 875 | 716 | 0.927905 | 6,117 | 8.54 | 1.55 | 0.30 | 0.39 |
| *J.tristani* | 2015 | 829 | 606 | 0.941242 | 5, 690 | 9.39 | 1.56 | 0.30 | 0.34 |

**Methods S4.** CarlaSeq (<https://github.com/molecularbiodiversity/carlaseq>) pipeline description of NGS data processing, assembly and assembly-based reference building using the target-enriched data sequenced. Raw sequencing data was treated by using a pipeline locally developed as follows:

**01-Trimming** Raw reads were trimmed or excluded whether Phred score < 33 using Trimmomatic 0.32^1^;

**02-Contaminant removal** Contaminant reads were identified using Kraken 0.10.4-beta^2^ and removed;

**03-Pairing** Sequences were paired and trimmed to 220 base pairs using Pear 0.9.4^3^;

**04-Demultiplex**: Paired reads were then demultiplexed and assigned to corresponding samples following the dual indexed adapters sequences;

**05-Fastq2fasta:** reads in fastq format, only from modern samples, were converted to fasta format and pooled into a single file;

**06-Assembling:** reads with 90% similarity were clustered to build representative loci centroids for further mapping using Vsearch 1.1.3 (<https://github.com/torognes/vsearch>);

**07-Filtering:** Cluster centroids built with less than 40 reads were removed;

**08-Paralogous filtering**: remaining centroids were de novo assembled with liberal similarity threshold (75%) using Geneious R7 software^4^ to check and remove putative similar centroids loci to ensure whether they were distinctive sequences or were related sequences derived from multiple loci, such as putative paralogous loci. Unclustered centroids were use to build a reference catalogue. Harvey et al.^5^ state that a more liberal similarity threshold (75%) enabled that few, but similar loci clusters (divergent alleles resulting from ‘over-splitting’ effect) to be assembled into one contig. It also enabled that several putative paralogous loci were collapsed into a single contig with high heterozigosity level and further discarded. Thus, contigs with more than 90% similarity across at least 55% fragment length were thereby discarded. High similar clusters with spurious alignments resulting from low complexity DNA sequences (comprised by mononucleotide repeats) were also discarded. Contigs with less than eight aligned clusters were synonymized into one single locus and joined to the unclustered loci to build the new assembly;

**09-Blast:** the probe set used for the enrichments was used to built a Blast database to find clusters that correspond to the targeted loci. Blast hits were annotated, extracted as new reference and splitted into mtDNA and nuclear DNA for further analysis.

**Table S5.** Blast analysis of ddRAD loci original probe set, the new reference assembly-based and its subset off-target loci based on *J. edwardsii* transcriptome (unpublished data).

| Query | Number of sequences | Blast hits |
| --- | --- | --- |
| ddRAD loci (nuclear) | 2,358 | 836 |
| New assembly (in- and off-target) | 5,940 | 4,368 |
| Only off-target loci form the new assembly | 1,773 | 925 |

**Table S6.** Non-parametric Spearman correlation matrix between sequencing yield, mapped reads, GC content, mean target coverage and Mapping quality in modern samples (N=40).

|  | Sequencing yield | Mapped  reads | Mean  coverage | Mapping quality | GC content |
| --- | --- | --- | --- | --- | --- |
| Sequencing yield | - |  |  |  |  |
| Mapped reads | .663** | - |  |  |  |
| Mean coverage | .631** | .997** | - |  |  |
| Mapping quality | .663** | .966** | .962** | - |  |
| GC content | .346* | -0.189 | -0.219 | -0.243 | - |

**. Correlation is significant at the 0.01 level (2-tailed).

*. Correlation is significant at the 0.05 level (2-tailed).

**Table S7.** Non-parametric Spearman correlation matrix between Year since sample, collection, GC content, A_260_/A_280_ ratio and Average heterozygous read rate in overall data including in modern and museum samples (N=79).

|  | Sequencing yield | Mapped  reads | Mean  coverage | GC content | Years since collection | A_260_/A_280_ ratio | Heterozygous read rate |
| --- | --- | --- | --- | --- | --- | --- | --- |
| Sequencing yield | - |  |  |  |  |  |  |
| Mapped reads | .933** | - |  |  |  |  |  |
| Mean coverage | .915** | .997** | - |  |  |  |  |
| GC content | .341** | .234* | 0.221 | - |  |  |  |
| Years since collection | -.418** | -.480** | -.489** | -.483** | - |  |  |
| A260/A280 | .569** | .611** | .622** | .482** | -.657** | - |  |
| Heterozygous read rate | -0.101 | -.230* | -.238* | -0.083 | 0.067 | -0.218 | - |

**. Correlation is significant at the 0.01 level (2-tailed).

*. Correlation is significant at the 0.05 level (2-tailed).

**Figure S8.** (**a**) Principal component analysis of sequencing yield, mapped reads, GC content, mean target coverage, year since collection, A_260_/A_280_ ratio and heterozygous read rate in overall *Jasus* samples (N=79). (**b**) Principal component analysis of sequencing yield, mapped reads, GC content, mean target coverage, year since collection, A260/A280 ratio and heterozygous read rate in historic samples (N=39). Squares denote museum samples and dots modern samples.

**
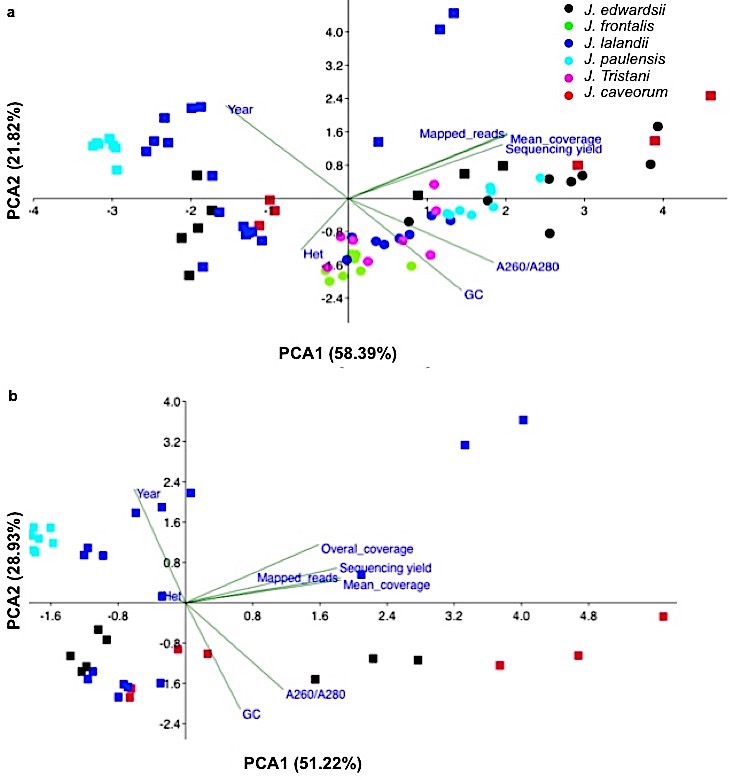
**

**Table S9.** GC content and read heterozygous rate differences between modern and historic samples within species.

| Species | Year | Variable | t | df | Sig | Mean Difference | Std. Error Difference | 95% Confidence  Lower | 95% Confidence  Upper |
| --- | --- | --- | --- | --- | --- | --- | --- | --- | --- |
| *J . edwardsii* | 1991 | GC content | 1.005 | 14 | 0.332 | -1.445 | 1.4382858 | -1.6398163 | 4.5298163 |
|  |  | Heterozygous reads rate | -1.491 | 7.095^a^ | 0.179 | 0.01039625 | 0.00697422 | -0.026843 | 0.0060505 |
| *J . paulensis* | 1967 | GC content | 21.279 | 8.802 ^a^ | 0.001** | -8.375 | 0.3935796 | 7.4815915 | 9.2684085 |
|  |  | Heterozygous reads rate | -2.837 | 7.415 ^a^ | 0.024* | 0.00880625 | 0.00310354 | -0.01606255 | -0.00154995 |
| *J .lalandii* | 1991 | GC content | 0.419 | 7.061 ^a^ | 0.688 | -0.61875 | 1.4784294 | -2.8710831 | 4.1085831 |
|  |  | Heterozygous reads rate | 0.316 | 14 | 0.756 | -0.00149875 | 0.00473608 | -0.00865913 | 0.01165663 |
| *J .lalandii* | 1967 | GC content | 10.123 | 7.321 ^a^ | 0.001** | -6.57 | 0.649046 | 5.0488006 | 8.0911994 |
|  |  | Heterozygous reads rate | 1.649 | 14 | 0.121 | -0.00476375 | 0.00288973 | -0.0014341 | 0.0109616 |

^a^Degrees of freedom adjusted using the Welch-Satterthwaite method.

**. Student’s t-test is significant at the 0.01 level (2-tailed).

*. Student’s t-test is significant at the 0.05 level (2-tailed).

**Figure S10.** PCA across and within species based on SNP genotypes, where deamination filter was applied. For each dataset, the sample passed filters and SNP pruning was adjusted to allow 0.10 maximum missing data per site and MAF < 0.05: **(a**) PCA across six *Jasus* species, 134 SNPs and 52 samples passed filters; (**b**) PCA of *J. lalandii*, 140 SNPs and 19 samples passed filter; (**c**) PCA of *J. edwardsii*, 665 variants and 12 samples passed filters; (**d**) PCA of *J. paulensis*, 106 SNPs and 14 samples passed filters.


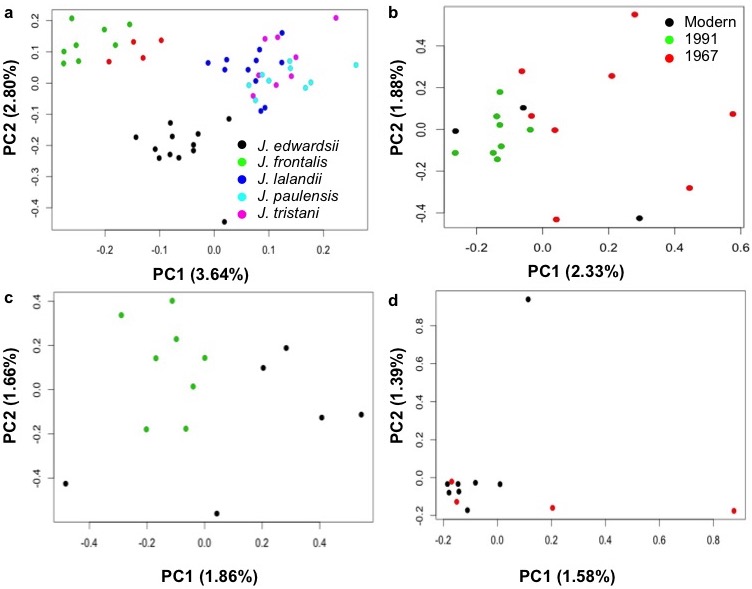


**Table S11.** Samples used in the target-capture experiment.

| **Samples** | **Species** | **Date** | **Country** | **Location** |
| --- | --- | --- | --- | --- |
| S1-BO128_jas1 | *J.edwardsii* | 2013 | Australia | East Tasmania/Bicheno |
| S3-BO156_jas1 | *J.edwardsii* | 2013 | Australia | East Tasmania/Bicheno |
| S2-BO159_jas1 | *J.edwardsii* | 2013 | Australia | East Tasmania/Bicheno |
| S4-MI014_jas1 | *J.edwardsii* | 2013 | Australia | East Tasmania/Maria Island |
| S5-MI015_jas1 | *J.edwardsii* | 2013 | Australia | East Tasmania/Maria Island |
| S6-MI044_jas1 | *J.edwardsii* | 2013 | Australia | East Tasmania/Maria Island |
| S7-BIC_jas1 | *J.edwardsii* | 2013 | Australia | East Tasmania/Bicheno |
| S8-MI014R_jas1 | *J.edwardsii* | 2013 | Australia | East Tasmania/Maria Island |
| S1-NIWA021_jas2 | *J.edwardsii* | 1991 | Australia | South Australia |
| S2-NIWA033_jas2 | *J.edwardsii* | 1991 | Australia | Tasman Sea |
| S3-NIWA034_jas2 | *J.edwardsii* | 1991 | Australia | Tasman Sea |
| S4-NIWA039_jas2 | *J.edwardsii* | 1991 | Australia | Tasman Sea |
| S5-NIWA040_jas2 | *J.edwardsii* | 1991 | Australia | Tasman Sea |
| S6-NIWA058_jas2 | *J.edwardsii* | 1991 | Australia | South Australia |
| S7-NIWA128_jas2 | *J.edwardsii* | 1991 | New Zealand | Off-east coast of New Zealand |
| S8-NIWA130_jas2 | *J.edwardsii* | 1991 | New Zealand | Off-east coast of New Zealand |
| S1-SAG001 | *S.verreauxii* | 2013 | Australia | Piccaninny, Tasmania |
| S2-SAG002 | *S.verreauxii* | 2013 | Australia | Piccaninny, Tasmania |
| S3-SAG003 | *S.verreauxii* | 2013 | Australia | Piccaninny, Tasmania |
| S4-SAG004 | *S.verreauxii* | 2013 | Australia | Bicheno, Tasmania |
| S5-SAG005 | *S.verreauxii* | 2013 | Australia | Bicheno, Tasmania |
| S6-SAG006 | *S.verreauxii* | 2013 | Australia | South West Rocks, New South Wales |
| S7-SAG007 | *S.verreauxii* | 2013 | Australia | Forster, New South Wales |
| S8-SAG004R | *S.verreauxii* | 2013 | Australia | Coffs Harbour, New South Wales |
| S1-SAG008 | *S.verreauxii* | 2013 | Australia | Coffs Harbour, New South Wales |
| S2-SAG009 | *S.verreauxii* | 2013 | Australia | Iluka, New South Wales |
| S3-SAG010 | *S.verreauxii* | 2013 | Australia | South West Rocks, New South Wales |
| S4-SAG011 | *S.verreauxii* | 2013 | New Zealand | 902 Statistical area of CRA1 |
| S5-SAG012 | *S.verreauxii* | 2013 | New Zealand | 902 Statistical area of CRA1 |
| S6-SAG013 | *S.verreauxii* | 2013 | New Zealand | 902 Statistical area of CRA1 |
| S7-SAG014 | *S.verreauxii* | 2013 | New Zealand | 902 Statistical area of CRA1 |
| S8-SAG016 | *S.verreauxii* | 2013 | New Zealand | 902 Statistical area of CRA1 |
| S1-SPA001 | *J.paulensis* | 2015 | Saint Paul Island | Pingouin |
| S2-SPA012 | *J.paulensis* | 2015 | Saint Paul Island | Pingouin |
| S3-SPA030 | *J.paulensis* | 2015 | Saint Paul Island | Pingouin |
| S4-SPA067 | *J.paulensis* | 2015 | Saint Paul Island | Pingouin |
| S5-SPA075 | *J.paulensis* | 2015 | Saint Paul Island | Pingouin |
| S6-SPA228 | *J.paulensis* | 2015 | Amsterdam Island | Del Cano |
| S7-SPA299 | *J.paulensis* | 2015 | Amsterdam Island | Del Cano |
| S8-SPA001R | *J.paulensis* | 2015 | Saint Paul Island | Pingouin |
| S1-TP155_spa2 | *J.paulensis* | 1967 | Saint Paul Island | - |
| S2-TP156_spa2 | *J.paulensis* | 1967 | Saint Paul Island | - |
| S3-TP163_spa2 | J.paulensis | 1967 | Saint Paul Island | - |
| S4-TP165_spa2 | *J.paulensis* | 1967 | Saint Paul Island | - |
| S5-TP166_spa2 | *J.paulensis* | 1967 | Saint Paul Island | - |
| S6-TP171_spa2 | *J.paulensis* | 1967 | Saint Paul Island | - |
| S7-TP172_spa2 | *J.paulensis* | 1967 | Saint Paul Island | - |
| S8-TP174_spa2 | *J.paulensis* | 1967 | Saint Paul Island | - |
| S1-TP005_cav | *J.caveorum* | 1995 | Foundation Seamounts | South East Pacific, S E of Pitcairn Island |
| S2-TP009_cav | *J.caveorum* | 1995 | Foundation Seamounts | South East Pacific, S E of Pitcairn Island |
| S4-TP025_cav | *J.caveorum* | 1995 | Foundation Seamounts | South East Pacific, S E of Pitcairn Island |
| S5-TP026_cav | *J.caveorum* | 1995 | Foundation Seamounts | South East Pacific, S E of Pitcairn Island |
| S6-TP028_cav | *J.caveorum* | 1995 | Foundation Seamounts | South East Pacific, S E of Pitcairn Island |
| S7-TP036_cav | *J.caveorum* | 1995 | Foundation Seamounts | South East Pacific, S E of Pitcairn Island |
| S8-TP005R_cav | *J.caveorum* | 1995 | Foundation Seamounts | South East Pacific, S E of Pitcairn Island |
| S1-TP041_fro1 | *J.frontalis* | 1967 | Chile | Islas Desventuradas |
| S2-TP044_fro1 | *J.frontalis* | 1967 | Chile | Islas Desventuradas |
| S3-TP045_fro1 | *J.frontalis* | 1967 | Chile | Islas Desventuradas |
| S4-TP051_fro1 | *J.frontalis* | 1967 | Chile | Islas Desventuradas |
| S5-TP053_fro1 | *J.frontalis* | 1967 | Chile | Islas Desventuradas |
| S6-TP059_fro1 | *J.frontalis* | 1967 | Chile | Islas Desventuradas |
| S7-TP063_fro1 | *J.frontalis* | 1967 | Chile | Islas Desventuradas |
| S8-TP063R_fro1 | *J.frontalis* | 1967 | Chile | Islas Desventuradas |
| S1-TP100_lal1 | *J.lalandii* | 1967 | South Africa | Cape Town |
| S2-TP102_lal1 | *J.lalandii* | 1967 | South Africa | Cape Town |
| S3-TP124_lal1 | *J.lalandii* | 1967 | South Africa | Cape Town |
| S4-TP126_lal1 | *J.lalandii* | 1967 | South Africa | Cape Town |
| S5-TP135_lal1 | *J.lalandii* | 1967 | South Africa | Cape Town |
| S6-TP140_lal1 | *J.lalandii* | 1967 | South Africa | Cape Town |
| S7-TP151_lal1 | *J.lalandii* | 1967 | South Africa | Cape Town |
| S8-TP151_lal1 | *J.lalandii* | 1967 | South Africa | Cape Town |
| S1-NIWA101_lal2 | *J.lalandii* | 1991 | South Africa | West coast |
| S2-NIWA102_lal2 | *J.lalandii* | 1991 | South Africa | West coast of southern Africa |
| S3-NIWA076_lal2 | *J.lalandii* | 1991 | South Africa | West coast of southern Africa |
| S4-NIWA083_lal2 | *J.lalandii* | 1991 | South Africa | South of southern Africa |
| S5-NIWA093_lal2 | *J.lalandii* | 1991 | South Africa | West coast of southern Africa |
| S6-NIWA104_lal2 | *J.lalandii* | 1991 | South Africa | West coast of southern Africa |
| S7-NIWA111_lal2 | *J.lalandii* | 1991 | South Africa | West coast of southern Africa |
| S8-NIWA116_lal2 | *J.lalandii* | 1991 | South Africa | West coast of southern Africa |
| S9-JLA079 | *J.lalandii* | 2015 | South Africa | Rocky Bank |
| S9-JLA113 | *J.lalandii* | 2015 | South Africa | Olifants_Bosch |
| S9-JLA136 | *J.lalandii* | 2015 | South Africa | Dassen Island |
| S9-JLA202 | *J.lalandii* | 2015 | South Africa | Knol |
| S9-JLA248 | *J.lalandii* | 2015 | South Africa | Kleinmond |
| S9-JLA265 | *J.lalandii* | 2015 | South Africa | Elands Bay |
| S9-JLA278 | *J.lalandii* | 2015 | South Africa | Port Nolloth |
| S9-JLA281 | *J.lalandii* | 2015 | South Africa | Port Nolloth |
| S10-JTR003 | *J.tristani* | 2015 | Off-coast South Africa | Tristan da Cunha |
| S10-JTR004 | *J.tristani* | 2015 | Off-coast South Africa | Tristan da Cunha |
| S10-JTR037 | *J.tristani* | 2015 | Off-coast South Africa | Gough Island |
| S10-JTR040 | *J.tristani* | 2015 | Off-coast South Africa | Gough Island |
| S10-JTR043 | *J.tristani* | 2015 | Off-coast South Africa | Nightingale Island |
| S10-JTR044 | *J.tristani* | 2015 | Off-coast South Africa | Nightingale Island |
| S10-JTR055 | *J.tristani* | 2015 | Off-coast South Africa | Inaccessible Island |
| S10-JTR056 | *J.tristani* | 2015 | Off-coast South Africa | Inaccessible Island |
| S10-JFR024 | *J.frontalis* | 2010 | Chile | Juan Fernandez Archipelago |
| S9-JFR032 | *J.frontalis* | 2010 | Chile | Juan Fernandez Archipelago |
| S9-JFR061 | *J.frontalis* | 2010 | Chile | Juan Fernandez Archipelago |
| S9-JFR088 | *J.frontalis* | 2010 | Chile | Juan Fernandez Archipelago |
| S9-JFR103 | J.frontalis | 2010 | Chile | Juan Fernandez Archipelago |
| S10-JFR124 | *J.frontalis* | 2010 | Chile | Juan Fernandez Archipelago |
| S10-JFR125 | *J.frontalis* | 2010 | Chile | Juan Fernandez Archipelago |
| S10-JFR036 | *J.frontalis* | 2010 | Chile | Juan Fernandez Archipelago |

**Table S12.** ddRAD libraries^6^ used for probe set design**.**

| **Sample** | **Species** | **Country** | **Location** |
| --- | --- | --- | --- |
| NZ3 | *S. verreauxi* | New Zealand | 902 Statistical area of CRA1 |
| NZ12 | *S. verreauxi* | New Zealand | 902 Statistical area of CRA1 |
| NZ19 | *S. verreauxi* | New Zealand | 902 Statistical area of CRA1 |
| NSW220 | *S. verreauxi* | Australia | Iluka, New South Wales |
| NSW271 | *S. verreauxi* | Australia | Coffs Harbour, New South Wales |
| T19 | *S. verreauxi* | Australia | Stanley Seaquarium, Tasmania |
| T27 | *S. verreauxi* | Australia | Stanley Seaquarium, Tasmania |
| NZ11rep | *S. verreauxi* | New Zealand | 902 Statistical area of CRA1 |
| T5 | *S. verreauxi* | Australia | Picaninny, Tasmania |
| NZ4 | *S. verreauxi* | New Zealand | 902 Statistical area of CRA1 |
| NZ13 | *S. verreauxi* | New Zealand | 902 Statistical area of CRA1 |
| NZ21 | *S. verreauxi* | New Zealand | 902 Statistical area of CRA1 |
| NSW230 | *S. verreauxi* | Australia | Iluka, New South Wales |
| NSW283 | *S. verreauxi* | Australia | Iluka, New South Wales |
| T20 | *S. verreauxi* | Australia | Stanley Seaquarium, Tasmania |
| T28 | *S. verreauxi* | Australia | Stanley Seaquarium, Tasmania |
| NSW220rep | *S. verreauxi* | Australia | Iluka, New South Wales |
| NZ5 | *S. verreauxi* | New Zealand | 902 Statistical area of CRA1 |
| NZ14 | *S. verreauxi* | New Zealand | 902 Statistical area of CRA1 |
| NZ38 | *S. verreauxi* | New Zealand | 902 Statistical area of CRA1 |
| NSW237 | *S. verreauxi* | Australia | Iluka, New South Wales |
| NSW284 | *S. verreauxi* | Australia | Iluka, New South Wales |
| T21 | *S. verreauxi* | Australia | Stanley Seaquarium, Tasmania |
| T29 | *S. verreauxi* | Australia | Stanley Seaquarium, Tasmania |
| T18rep | *S. verreauxi* | Australia | Stanley Seaquarium, Tasmania |
| NZ7 | *S. verreauxi* | New Zealand | 902 Statistical area of CRA1 |
| NZ15 | *S. verreauxi* | New Zealand | 902 Statistical area of CRA1 |
| NSW163 | *S. verreauxi* | Australia | South West Rocks, New South Wales |
| NSW249 | *S. verreauxi* | Australia | Coffs Harbour, New South Wales |
| NSW333 | *S. verreauxi* | Australia | South West Rocks, New South Wales |
| T22 | *S. verreauxi* | Australia | Stanley Seaquarium, Tasmania |
| T32 | *S. verreauxi* | Australia | Stanley Seaqurium, Tasmania |
| NZ8 | *S. verreauxi* | New Zealand | 902 Statistical area of CRA1 |
| NZ16 | *S. verreauxi* | New Zealand | 902 Statistical area of CRA1 |
| NSW175 | *S. verreauxi* | Australia | South West Rocks, New South Wales |
| NSW253 | *S. verreauxi* | Australia | Coffs Harbour, New South Wales |
| NSW337 | *S. verreauxi* | Australia | South West Rocks, New South Wales |
| T23 | *S. verreauxi* | Australia | Stanley Seaquarium, Tasmania |
| T33 | *S. verreauxi* | Australia | Stanley Seaquarium, Tasmania |
| JB1 | *S. verreauxi* | Australia | Jervis Bay, New South Wales |
| NZ10 | *S. verreauxi* | New Zealand | 902 Statistical area of CRA1 |
| NZ17 | *S. verreauxi* | New Zealand | 902 Statistical area of CRA1 |
| NSW194 | *S. verreauxi* | Australia | Forster, New South Wales |
| NSW254 | *S. verreauxi* | Australia | Coffs Harbour, New South Wales |
| T16 | *S. verreauxi* | Australia | Stanley Seaquarium, Tasmania |
| T24 | *S. verreauxi* | Australia | Stanley Seaquarium, Tasmania |
| T37 | *S. verreauxi* | Australia | Stanley Seaquarium, Tasmania |
| NZ20 | *S. verreauxi* | New Zealand | 902 Statistical area of CRA1 |
| NZ11 | S. verreauxi | New Zealand | 902 Statistical area of CRA1 |
| NZ18 | *S. verreauxi* | New Zealand | 902 Statistical area of CRA1 |
| NSW201 | *S. verreauxi* | Australia | Forster, New South Wales |
| NSW263 | *S. verreauxi* | Australia | Iluka, New South Wales |
| T18 | *S. verreauxi* | Australia | Stanley Seaquarium, Tasmania |
| T26 | *S. verreauxi* | Australia | Stanley Seaquarium, Tasmania |
| T38 | *S. verreauxi* | Australia | Stanley Seaquarium, Tasmania |
| 427354 | *J. edwardsii* | Australia | Southwest Tasmania/Maatsuyker Island |
| 427367 | *J. edwardsii* | Australia | Southwest Tasmania/Maatsuyker Island |
| 427369 | *J. edwardsii* | Australia | Southwest Tasmania/Maatsuyker Island |
| 427381 | *J. edwardsii* | Australia | Southwest Tasmania/Maatsuyker Island |
| Auckland | *J. edwardsii* | New Zealand | New Zealand/Auckland |
| BFC1 | *J. edwardsii* | Australia | South Australia/Blackfellow's Caves |
| BFC3 | *J. edwardsii* | Australia | South Australia/Blackfellow's Caves |
| BFC5 | *J. edwardsii* | Australia | South Australia/Blackfellow's Caves |
| BFC5 | *J. edwardsii* | Australia | South Australia/Blackfellow's Caves |
| BFC6 | *J. edwardsii* | Australia | South Australia/Blackfellow's Caves |
| BR001 | *J. edwardsii* | Australia | East Tasmania/Bruny Island |
| BR002 | *J. edwardsii* | Australia | East Tasmania/Bruny Island |
| BR003 | *J. edwardsii* | Australia | East Tasmania/Bruny Island |
| BR004 | *J. edwardsii* | Australia | East Tasmania/Bruny Island |
| BR005 | *J. edwardsii* | Australia | East Tasmania/Bruny Island |
| FS10 | *J. edwardsii* | Australia | East Tasmania/The Friars |
| FS11 | *J. edwardsii* | Australia | East Tasmania/The Friars |
| FS13 | *J. edwardsii* | Australia | East Tasmania/The Friars |
| FS6 | *J. edwardsii* | Australia | East Tasmania/The Friars |
| FS7 | *J. edwardsii* | Australia | East Tasmania/The Friars |
| FS8 | *J. edwardsii* | Australia | East Tasmania/The Friars |
| FS9 | *J. edwardsii* | Australia | East Tasmania/The Friars |
| MAA11 | *J. edwardsii* | Australia | Southwest Tasmania/Maatsuyker Island |
| MAA12 | *J. edwardsii* | Australia | Southwest Tasmania/Maatsuyker Island |
| MAA13 | *J. edwardsii* | Australia | Southwest Tasmania/Maatsuyker Island |
| MAA14 | *J. edwardsii* | Australia | Southwest Tasmania/Maatsuyker Island |
| MAA15 | *J. edwardsii* | Australia | Southwest Tasmania/Maatsuyker Island |
| MAA7 | *J. edwardsii* | Australia | Southwest Tasmania/Maatsuyker Island |
| MMS12 | *J. edwardsii* | Australia | Victoria/Merri Marine Sanctuary |
| MMS13 | *J. edwardsii* | Australia | Victoria/Merri Marine Sanctuary |
| MMS14 | *J. edwardsii* | Australia | Victoria/Merri Marine Sanctuary |
| MMS15 | *J. edwardsii* | Australia | Victoria/Merri Marine Sanctuary |
| MMS16 | *J. edwardsii* | Australia | Victoria/Merri Marine Sanctuary |
| MMS21 | *J. edwardsii* | Australia | Victoria/Merri Marine Sanctuary |
| MMS22 | *J. edwardsii* | Australia | Victoria/Merri Marine Sanctuary |
| Tonga | *J. edwardsii* | New Zealand | New Zealand/Tonga |
| TX19 | *J. edwardsii* | Australia | East Tasmania/Tinderbox |
| TX3 | *J. edwardsii* | Australia | East Tasmania/Tinderbox |
| TX30 | *J. edwardsii* | Australia | East Tasmania/Tinderbox |
| TX31 | *J. edwardsii* | Australia | East Tasmania/Tinderbox |
| TX35 | *J. edwardsii* | Australia | East Tasmania/Tinderbox |
| TX36 | *J. edwardsii* | Australia | East Tasmania/Tinderbox |
| TX4 | *J. edwardsii* | Australia | East Tasmania/Tinderbox |
| TX48 | *J. edwardsii* | Australia | East Tasmania/Tinderbox |

**Table S13.** Protocol of genomic library preparation prior hybrid capture, adapted from Rohland and Reich (2012)^7^. Rohland and Reich protocol enabled 192 libraries to be processed in six hours hands-on time with a rough cost of $14 per library. In the present study we omitted the initial size-selection as suggested by Mamanova et al. (2010)^8^ and performed a pre-capture multiplexing previous described in Shearer et al.^9^ (2012). Although these modiﬁcations together reduced costs by at least 38%, the decrease in capture efﬁciency has been reported but did not negatively affect variant detection^9^ even with low-coverage librarie^10^ in other studies.

| **Step** | **Description** | **Volume** |
| --- | --- | --- |
| Sonication DNA shearing | 25 cycles: 90'' on/30'' off | 60 μL |
| 5' e 3' end-repair | Quick blunting kit (NEB)* | 65 μL |
| Adaptor and barcode ligation | Quick ligation kit (NEB)*; 2ul adaptor-P5 (200 μM); 2 μL adaptor-P7 (200 μM). | 83.5 μL |
| Purification with magnetic beads | AMPure beads XP 1.0X. Re-suspended in mM Tris-HCl | 17 μL |
| Nick fill-in | 8U Bst DNA Polymerase (NEB) | 25 μL |
| Purification with magnetic beads | AMPure beads XP 1.2X. Re-suspended in 10 mM Tris-HCl | 17 μL |
| Indexing PCR | *NEBNext Q5 Hot Start HiFi PCR Master Mix*; 1 indexed primer adaptor (10 μM); 1 μL universal primer P1Illumina (10 μM) - 18 cycles | 25 μL |
| Library validation | *NEBNext® Library Quant Kit* for Illumina | 20 μL |
| Pre-capture libraries multiplexing | 100ng-500ng DNA | - |

**Table S14.** Multiplexed libraries *prior* hybridization capture reaction. Negative controls did not reveal detectable amounts of DNA, still they were included in a separate library and sequenced.

| Target-capture  pool | Species | N | Captured product (ng/ul) | PCR-product (ng) | Seq. pooling volume  (ul) | Total amount of DNA  (ng) |
| --- | --- | --- | --- | --- | --- | --- |
| 1 | *J. edwardsii* | 16 | 0.157 | 5.5 | 3.62 | 20 |
| 2 | *S. verreauxi* | 16 | 0.211 | 10.8 | 1.85 | 20 |
| 3 | *J. paulensis* | 16 | 0.216 | 12.8 | 1.56 | 20 |
| 4 | *J.caveorum/J. frontalis* | 16 | 0.174 | 12.1 | 1.65 | 20 |
| 6 | *J. lalandii* | 16 | 0.06 | 9.59 | 2.09 | 20 |
| 8 | *J.tristani/ J.lalandii* | 16 | 0.168 | 2.6 | 7.69 | 20 |
| 9 | *J. frontalis* | 8 | 0.81 | 4.99 | 2.00 | 10 |

| **Step** | **Description** | **Volume** |
| --- | --- | --- |
| Library concentration | Vacuum centrifuge; 10 min. | 7 μL |
| Hybridization | *Mybaits®* customized biotinylated probes; 65 °C for 16 hours. | 25 μL |
| Capture | Streptavidin DyNA beads, capture buffer, 55 °C for 5 minutes. |  |
| Enrichment/Washing | Beads washed 4x with washing buffer. |  |
| Amplification | *NEBNext Q5 Hot Start HiFi PCR Master Mix*; 1 uL universal primerP1 Illumina (10 μL); 1 μL universal primer P2 Illumina (10 μL) | 25 μL |

**Table S15.** Experimental workflow of library enrichment protocol of pre-multiplexed genomic libraries using Mybaits® custom probes (Mycroarray).

# References

1. Bolger, a. M., Lohse, M. & Usadel, B. Trimmomatic: a flexible trimmer for Illumina sequence data. *Bioinformatics* **30,** 2114–2120 (2014).

2. Wood, D. E. & Salzberg, S. L. Kraken: ultrafast metagenomic sequence classification using exact alignments. *Genome Biol.* **15,** R46 (2014).

3. Zhang, J., Kobert, K., Flouri, T. & Stamatakis, A. PEAR: A fast and accurate Illumina Paired-End reAd mergeR. *Bioinformatics* **30,** 614–620 (2014).

4. Olsen, C. Geneious R7: A Bioinformatics Platform for Biologists. *Plant Anim. Genome XXII Conf.* (2014).

5. Harvey, M. G. *et al.* Similarity threshholds used in short read assembly reduce the comparability of population histories across species. *PeerJ* **PrePrints,** 1–34 (2015).

6. Villacorta-Rath, C. *et al.* Outlier SNPs enable food traceability of the southern rock lobster, Jasus edwardsii. *Mar. Biol.* **163,** 223 (2016).

7. Rohland, N. & Reich, D. Cost-effective, high-throughput DNA sequencing libraries for multiplexed target capture. *Genome Res.* **22,** 939–946 (2012).

8. Mamanova, L. *et al.* Target-enrichment strategies for next-generation sequencing. *Nat. Methods* **7,** 111–8 (2010).

9. Shearer, A. E. *et al.* Pre-capture multiplexing improves efficiency and cost-effectiveness of targeted genomic enrichment. *BMC Genomics* **13,** 618 (2012).

10. Hancock-Hanser, B. L. *et al.* Targeted multiplex next-generation sequencing: Advances in techniques of mitochondrial and nuclear DNA sequencing for population genomics. *Mol. Ecol. Resour.* **13,** 254–268 (2013).
